# Do Kinematics or Muscle Function During Sit-to-Stand Change Following a Primary Total Knee Arthroplasty?

**DOI:** 10.1007/s10439-025-03782-3

**Published:** 2025-07-21

**Authors:** Kathryn S. Blessinger, Sarah A. Roelker, Reese A. Lloyd, Laura C. Schmitt, Ajit M. W. Chaudhari, Robert A. Siston

**Affiliations:** 1 Department of Mechanical and Aerospace Engineering, The Ohio State University, Columbus, OH USA; 2 Department of Kinesiology, University of Massachusetts Amherst, Amherst, MA USA; 3 Department of Biomedical Engineering, The Ohio State University, Columbus, OH USA; 4 School of Health and Rehabilitation Sciences, The Ohio State University, Columbus, OH USA; 5 Sports Medicine Research Institute, The Ohio State University Wexner Medical Center, Columbus, OH USA; 6 Division of Physical Therapy, School of Health and Rehabilitation Sciences, The Ohio State University, Columbus, OH USA; 7 Department of Orthopaedics, The Ohio State University, Columbus, OH USA; 8 Department of Mechanical and Aerospace Engineering, The Ohio State University, N350 Scott Laboratory, 201 W19th Ave, Columbus, OH 43210 USA

**Keywords:** Musculoskeletal model, OpenSim, Muscle-driven simulation, Sit-to-stand, Total knee arthroplasty

## Abstract

Sit-to-stand (STS) transfer is essential for independent living and mobility in daily life but challenging for individuals before and after total knee arthroplasty (TKA). It remains unclear whether deficits in individual muscle function contribute to the lack of improvement in STS performance following TKA. The objective of this study was to determine whether lower extremity kinematics, muscle function during STS (quantified by muscle forces and muscle contributions to acceleration), and knee extension strength change following TKA. Dynamic musculoskeletal simulations of STS were performed for seven individuals before and six months after surgery. Computed muscle control and induced acceleration analysis were used to determine individual muscle forces and contributions to center of mass acceleration, respectively. Overall kinematic strategy did not largely change following TKA, and we found no change in muscle forces, muscle contributions, or knee extension strength following TKA, suggesting a consistent movement strategy to compensate for persistent quadriceps weakness. Both before and after TKA, the psoas, gluteus medius, vastus medialis and lateralis, and soleus produced the largest forces, while the gluteus maximus and biceps femoris produced smaller forces. Large braking contributions to acceleration from several muscles and small gluteus maximus contributions to forward and upward acceleration observed at both time points may contribute to the persistent functional performance deficits often observed in patients with a TKA. These findings demonstrate that overall movement strategy during STS (quantified by kinematics and muscle function) and knee extension strength did not change within six months following TKA, which may inform post-operative rehabilitation programs.

**Supplementary Information:**

The online version contains supplementary material available at 10.1007/s10439-025-03782-3.

## Introduction

Rising from a chair, clinically known as the sit-to-stand (STS) transfer, is a common and demanding task performed more than 45 times per day [1]. Being able to perform STS is essential for independent living and mobility in daily life [2]. The STS transfer is particularly challenging for individuals with lower extremity muscle weakness [3] and knee pathologies, including individuals with knee osteoarthritis (KOA), even after undergoing a total knee arthroplasty (TKA) [4, 5]. While TKA aims to improve functional limitations so that individuals with end-stage KOA can better perform activities of daily living, approximately 20% of patients experience postoperative dissatisfaction and continued mobility limitations [6]. These mobility deficits are reflected in a lack of clinically meaningful improvements in functional performance, particularly within the first six months of recovery following TKA [7]. Specifically during STS, approximately 31% of TKA patients report continued difficulty when rising from a chair [8].

Functional limitations during STS are present both before and after TKA, including slow STS speeds and altered movement strategies [4, 9–12]. Altered movement strategies that persist after a primary TKA include greater forward trunk flexion to reduce the required knee extension moment [11, 13, 14], smaller knee extension range of motion [15, 16], weightbearing asymmetry to shift weight away from the involved limb [11, 17], and co-activation of muscles spanning the knee during STS [15, 18]. Previous experimental work has demonstrated that patients up to one year after a TKA exhibit altered kinematic, kinetic, and neuromuscular control strategies during STS, when compared to healthy controls, to compensate for weakened quadriceps [13] and to provide knee joint stability [18]. These strategies do not largely improve from preoperative conditions [11, 14, 15]. However, experimental techniques alone do not fully explain the underlying function of individual muscles during dynamic tasks, like STS [19].

Musculoskeletal simulations have the ability to investigate the role of individual muscle forces produced during dynamic tasks and how they contribute to accelerating the center of mass (COM) [19, 20]. Understanding how muscles contribute to accelerating the COM up and forward during STS may better inform rehabilitation programs and improve patients’ ability to perform STS, as a muscle’s contribution to acceleration can be counterintuitive to its anatomical function or skeletal alignment [21]. Previous studies have characterized muscle function during STS by examining muscle forces and muscle contributions to acceleration in healthy, young adults [22, 23]. Caruthers et al. [22] found the quadriceps to be key contributors in lifting the COM vertically and slowing forward progression of the COM when performing STS. Additionally, when the quadriceps were virtually weakened in a series of simulations, reflecting the 20–40% quadriceps strength deficits typically present before and after TKA [24–26], Caruthers et al. [23] identified muscles that can compensate for this weakness in order to maintain the kinematics of healthy, young adults performing STS, including increasing force production of the hip flexors and decreasing the force production of the biceps femoris, gluteus maximus, and gastrocnemii. However, it remains unclear how muscle function during STS changes from before to after a primary TKA, particularly when the kinematic strategy differs from that of healthy, young adults.

Therefore, the purposes of this study were to (1) compare sagittal plane kinematics during STS in individuals with end-stage KOA to those exhibited by the same individuals 6 months after undergoing a primary TKA, (2a) compare involved limb lower extremity muscle forces and (2b) interlimb muscle force asymmetry before and after a primary TKA, (3) compare involved limb muscle contributions to COM acceleration before and after a primary TKA. We hypothesized that after TKA, individuals would demonstrate: (1) decreased forward lumbar flexion, anterior pelvic tilt, and involved limb hip flexion and dorsiflexion, but increased involved limb knee extension following TKA; (2a) increased quadriceps muscle forces in the involved limb and (2b) decreased quadriceps interlimb muscle force asymmetry during STS; and (3) increased muscle contributions to COM accelerations from the quadriceps in the involved limb. As a secondary analysis to aid in interpretation of primary hypothesis testing, (4) we examined differences in isometric knee extension strength before and after TKA and (4) hypothesized that individuals would demonstrate increased knee extension strength following TKA.

## Materials and Methods

### Experimental Data

Seven individuals with predominantly medial compartment KOA, who were scheduled to undergo a primary posterior-stabilizing TKA (Table 1), provided written informed consent and enrolled in a prospective observational cohort study. Participants underwent standard of care postoperative treatment following TKA. The individuals analyzed in the current study are a subset of that reported in Freisinger et al. [27], Chaudhari et al. [28], and Koehn et al. [29]. Kinematic, kinetic, and electromyography (EMG) data were collected approximately one month prior to surgery and six months after surgery while the participants performed four chair rises. Participants were instructed to sit down from a standing position and then rise from the chair, standing completely in between each transfer, with arms crossed at the chest. All participants performed each chair rise from an armless, hard-backed, 55.2 cm chair. Whole-body kinematics were captured by a 10-camera motion capture system at 150 Hz (MX-F40, Vicon, Oxford, UK) using a modified point cluster technique marker set [27, 30]. Ground reaction forces (GRF) were recorded at 1500 Hz from two force plates (Bertec Corp, Columbus, OH), one placed under each foot; no part of the chair touched either force plate. Surface EMG data were collected at 1500 Hz (Noraxon, Scottsdale, AZ; Vermed, Buffalo, NY) bilaterally on rectus femoris, vastus lateralis, vastus medialis, biceps femoris, semitendinosus, lateral gastrocnemius, medial gastrocnemius, and soleus. The EMG data were demeaned, bandpass filtered (between 50 Hz and 300 Hz), rectified, and smoothed using a 6 Hz lowpass filter [29]. One representative chair rise was analyzed for each participant at each time point (pre- and post-TKA) and was chosen by the quality of marker, force plate, and EMG data. The STS transfer was divided into 3 phases (forward leaning, momentum transfer, and extension), each distinguished by kinematic data (Fig S1) [31]. Involved limb knee extension strength was assessed at each time point with an electromechanical dynamometer (Biodex System 3, Biodex Medical Systems; Shirley, NY) during a maximal voluntary isometric contraction with the involved knee flexed at 60 degrees [27, 28].

**Table 1:** Subject demographics before and after a total knee arthroplasty (TKA)

	Sex	Age (years)	Mass (kg)	Height (m)	Body Mass Index (kg/m^2^)
Before TKA	4F, 3 M	59.4 ± 8.52	88.0 ± 6.38	1.69 ± 0.08	31.0 ± 3.14
After TKA	4F, 3 M	60.0 ± 8.59	88.4 ± 9.66	1.69 ± 0.08	30.8 ± 3.12

### Musculoskeletal Model and Simulations

A modified Bosch et al. musculoskeletal model [32] was used to conduct dynamic, muscle-driven simulations in OpenSim 4.4 [19] for 1 chair rise per participant at each time point (before and after TKA). The Bosch 2022 model was chosen as a base model for this study, as it has been previously validated for investigating lower extremity muscles during chair rise motions in healthy individuals, and it includes improved estimates for knee extensor moment arms [32]. However, to better model the active and passive knee joint kinematics demonstrated by the KOA and TKA populations [33–35], we modified the Bosch model to include two additional degrees of freedom at the knee: knee adduction/abduction and knee internal/external rotation. Additionally, we modified the muscle attachment points of the hip abductor muscles and calibrated each muscle’s passive force-length curve to more closely match experimental data, as done in Uhlrich et al. [36]; more details on these modifications are available in the Supplementary Materials (Tables S1–3, Figs S2–3). The final, generic musculoskeletal model utilized in the current study contains 25 degrees of freedom, 80 musculotendon actuators on the lower extremities, and 3 ideal torque actuators on the torso.

Dynamic, musculoskeletal simulations were conducted in OpenSim 4.4 [19] to compute lower extremity kinematics and to estimate individual muscle function (muscle forces and muscle contributions to COM accelerations), using the following simulation pipeline. The generic musculoskeletal model was first scaled to the anthropometrics of each participant’s static calibration trial, and then inverse kinematics was used to solve for the model’s joint angles throughout the dynamic STS trial, using a least squares approach to minimize the difference between experimental marker data and corresponding virtual model markers, with the RMS marker error not exceeding 3.5 cm [19, 20]. The residual reduction algorithm (RRA) was used to adjust the model’s mass properties in order to be more dynamically consistent with experimental GRF data [19]. Inverse dynamics (ID) was used to determine joint torques on the RRA-adjusted model [19], and computed muscle control (CMC) was used to further resolve the net joint torques into individual muscle forces during each chair rise [37, 38]. An initial run of CMC determined initial simulated muscle activations for use in EMG normalization, where each muscle’s EMG data were normalized to the corresponding peak experimental muscle activation within each STS trial, and then multiplied by the initial peak simulated activation [22]. In a second run of CMC, muscle excitation ranges were then constrained using muscle-specific and participant-specific windows about the corresponding experimental EMG data [39].

**Fig 1.**
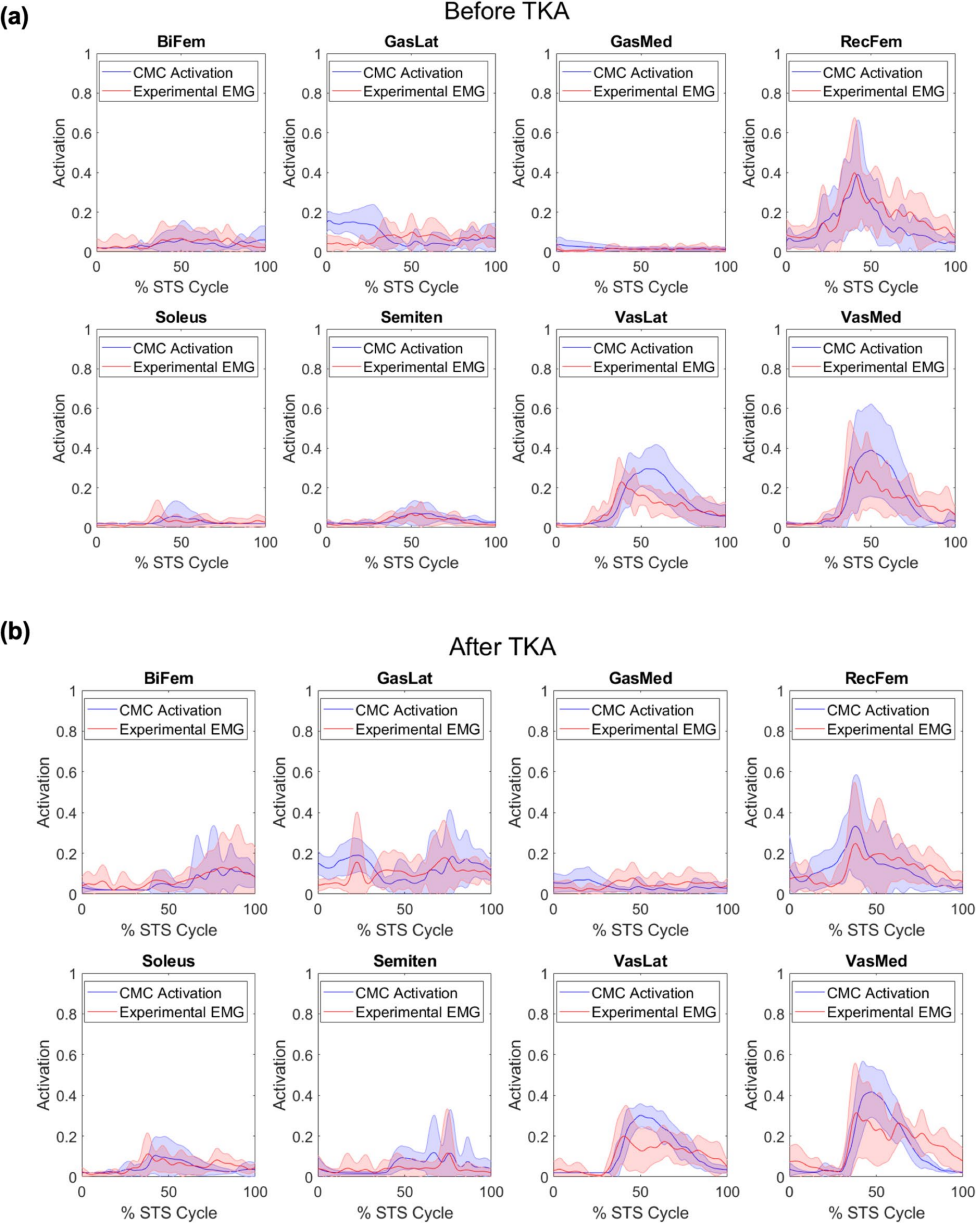
Mean ± standard deviation of simulated muscle activations (blue) from computed muscle control (CMC) and experimental electromyography (EMG) (red) for the involved limb of 7 participants (**a**) before a total knee arthroplasty (TKA) and (**b**) after TKA, over the sit-to-stand (STS) cycle. Abbreviations: BiFem, biceps femoris; GasLat, lateral gastrocnemius; GasMed, medial gastrocnemius; RecFem, rectus femoris; Semiten, semitendinosus; VasLat, vastus lateralis; VasMed, vastus medialis

**Fig 2.**
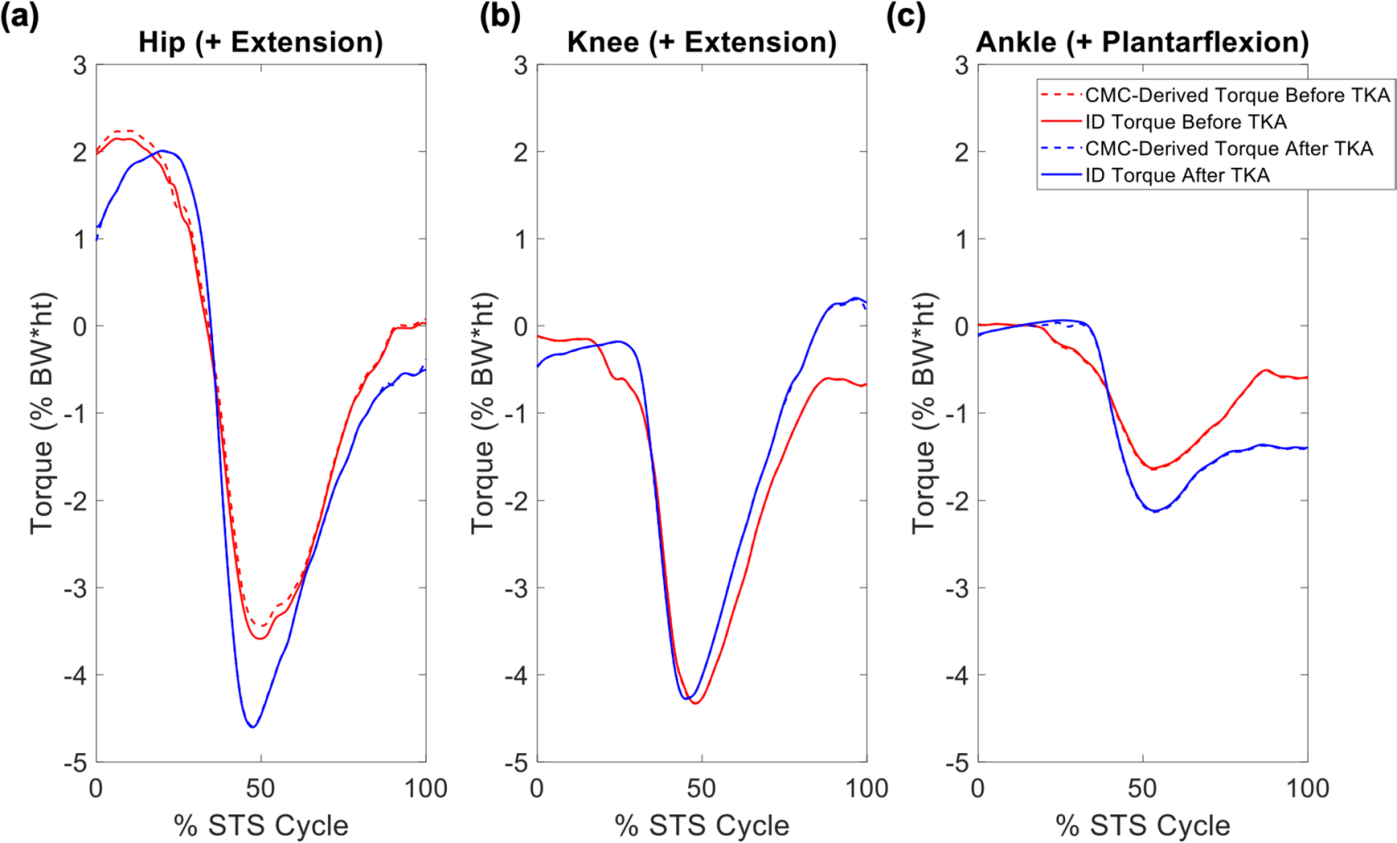
Average sagittal plane (**a**) hip, (**b**) knee, and (**c**) ankle joint torques from inverse dynamics (solid lines) and the sum of the computed muscle control (CMC)-derived muscle torques (dashed lines) for the involved limb of all 7 participants before a total knee arthroplasty (TKA) (red) and after TKA (blue), over the sit-to-stand (STS) cycle. All torques are external

Simulated muscle activations were quality checked to ensure that simulated muscle activations exhibited similar timing and magnitude to available EMG data [20], where simulated activations from the final run of CMC were used in the final EMG normalization (Fig 1) [22]. To verify that the simulation was muscle-actuated in the sagittal plane, we ensured that the sagittal plane hip, knee, and ankle ID joint torques (normalized to %BW*ht) matched those calculated from CMC by summing muscle forces, multiplied by their respective moment arms, for each joint (Fig 2) [22, 40, 41].

An induced acceleration analysis (IAA) in OpenSim computed the potential of each muscle (per unit of force) to accelerate the COM vertically and horizontally [21, 42–44]. A rolling-on-surface constraint was used for both feet, with the ground as the surface body and the calcaneus as the rolling body [22, 45]. To obtain individual muscle contributions to vertical and horizontal COM acceleration, muscle potentials (obtained from IAA) were multiplied by the corresponding muscle force (obtained from CMC) at each time step of the STS trial [22, 42, 43]. We used definitions for vertical and horizontal COM accelerations and horizontal COM braking that are consistent with other STS literature [22] (Supplementary Materials: Fig S4). Vertical COM braking is a new term that has not been previously defined, as limited work has examined muscle contributions during STS in orthopaedic populations; this new definition is shown in Figure S4 and represents the COM deceleration (braking) in the vertical direction.

### Data Analysis

Separate general linear models (GLMs) and paired t-tests allowed us to compare kinematics, muscle forces, interlimb muscle force asymmetry, and muscle contributions between time points. For all GLMs, participant was included as a random factor, and interactions between fixed factors, which are listed below for each hypothesis, were included in each GLM (Supplementary Material: Table S4). Post hoc Tukey pairwise comparisons were used as appropriate. All statistical tests were performed in Minitab Statistical Software (Minitab Inc, State College, PA) at a significance level of α = 0.05.

For hypothesis 1, peak hip, knee, and ankle (HKA) sagittal plane angles from each limb, within each phase of the STS cycle, were compared between time points using separate GLMs. Limb, phase, and time (before or after TKA) were examined as fixed factors. In the sagittal plane, peak lumbar flexion angles and peak anterior/posterior pelvic tilt angles, within each phase of the STS cycle, were compared between time points using separate GLMs with phase and time as fixed factors. Additionally, initial sagittal plane HKA kinematics (angles at 0% of the STS cycle) from each limb were compared between time points and limbs using separate GLMs with limb and time as fixed factors. Moreover, when response variables were not measured across the sit-to-stand cycle (i.e., phase not needed as a fixed factor), and when we did not have response variable data for both limbs (i.e., limb not needed as a fixed factor), we used paired-samples t testing to assess for differences between timepoints in the response variables. Therefore, initial sagittal plane lumbar flexion angle, initial sagittal plane pelvic tilt angle, and initial involved limb anteroposterior foot placement (Supplementary Material: Fig S5) were compared between time points using paired-samples t tests.

For hypothesis 2a and 3, peak involved limb (2a) muscle forces and (3) muscle contributions within each phase were compared between time points using separate GLMs with muscle, phase, and time as fixed factors. For hypothesis 2b, we defined asymmetry in muscle forces as the percent difference between the involved and uninvolved limbs’ peak muscle forces within each phase [22]. Interlimb muscle force asymmetry was compared between time points using a GLM with muscle, phase, and time as fixed factors. For hypothesis 4, involved limb knee extension strength was compared between time points using paired-samples t tests, since phase and limb were not applicable fixed factors for the involved limb knee extension strength data.

## Results

### Hypothesis 1: Sagittal Plane Kinematics

**Fig 3.**
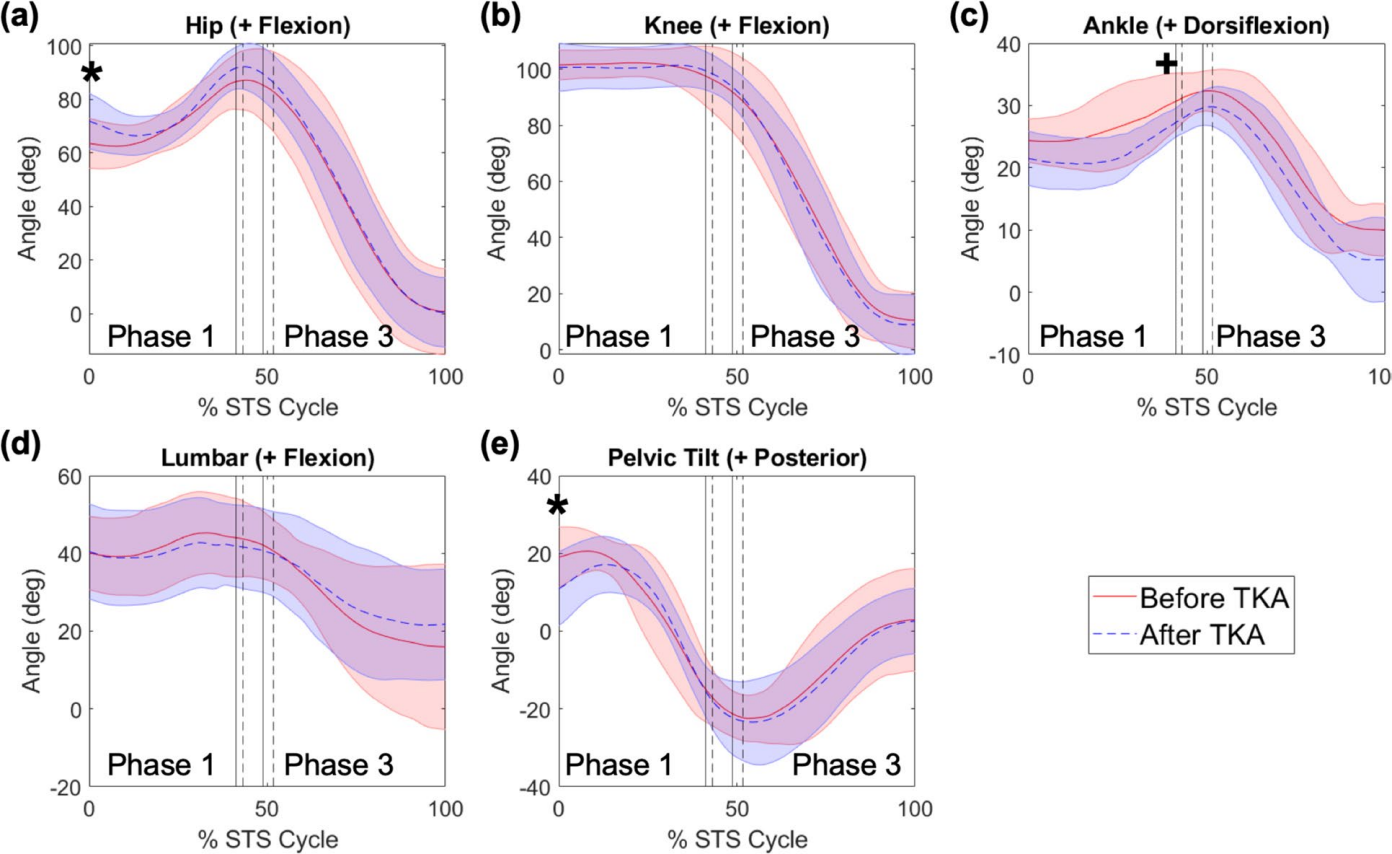
Mean ± standard deviation of involved limb sagittal plane (**a**) hip, (**b**) knee, (**c**) ankle angles and sagittal plane (**d**) lumbar and (**e**) pelvic tilt angles before (red) and after (blue) total knee arthroplasty (TKA), over the sit-to-stand (STS) cycle. Phases of the STS cycle are indicated by black vertical lines, with solid lines indicating before TKA and dashed indicating after TKA. In (**a**) and (**e**), * indicates significant difference in initial hip and initial pelvic tilt angles before and after TKA. In (**c**), + indicates significant difference in peak ankle angle during phase 1 before and after TKA

There was no significant main effect of time (before versus after TKA) on the sagittal plane peak lumbar angles (*p* = 0.570), peak anterior/posterior pelvic tilt angles (*p* = 0.346), peak hip angles (*p* = 0.062), peak knee angles (*p* = 0.087), initial knee angle (*p* = 0.425), and initial ankle angle (*p* = 0.102). There were no significant interactions for any of the kinematics GLMs (*p* ≥ 0.137). Additionally, there was no significant difference in initial lumbar angle (*p* = 0.885) or initial involved limb foot placement (*p* = 0.365; Supplementary Material: Table S5) between time points. However, there was a significant main effect of time on the peak ankle angles (*p* < 0.001), and there were significant differences between time points in the initial pelvic tilt angle (*p* = 0.021) and initial hip angle (*p* < 0.001). During phase 1 in the involved limb, individuals demonstrated decreased peak dorsiflexion angles after surgery (before: 32.1 ± 4.4°, after: 27.6 ± 2.7°; *p* = 0.013; Fig 3c). After TKA, individuals demonstrated decreased initial posterior pelvic tilt (before: 19.0 ± 7.8°, after: 10.9 ± 9.5°, *p* = 0.021, Fig 3e) and increased initial hip flexion in both the involved (before: 63.5 ± 9.3°, after: 71.8 ± 10.3°; *p* = 0.015; Fig 3a) and uninvolved limbs (before: 63.5 ± 8.6°, after: 70.6 ± 9.1°; *p* = 0.042; Supplementary Material: Fig S6a). There were no significant main effects of limb on any of the kinematic measures except for peak ankle angles (*p* = 0.035). However, post hoc analyses yielded no significant differences in peak ankle angle between limbs within the same time point and phase (Fig 3, Fig S6a, *p* > 0.857).

### Hypothesis 2: Muscle Forces

**Fig 4.**
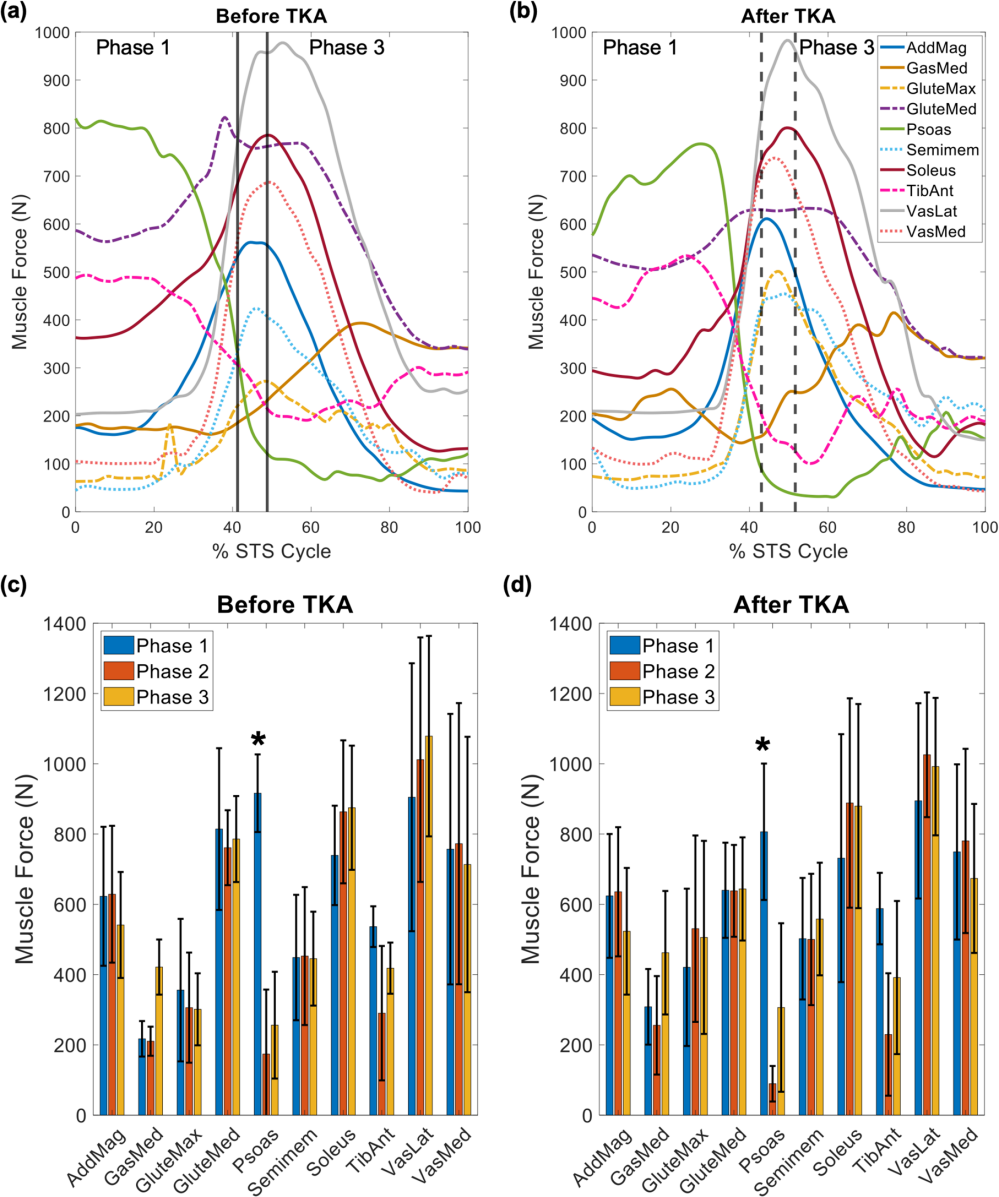
(**a**, **b**) Average muscle forces over the sit-to-stand (STS) cycle, for the involved limb (**a**) before total knee arthroplasty (TKA) and (**b**) after TKA. Vertical lines denote phases of the STS cycle before (solid) and after (dashed) TKA. All other muscles exhibited peak muscle forces of magnitude less than 400 N both before and after TKA. (**c**, **d**) Average peak muscle forces across all 7 participants for each phase of the STS cycle, (**c**) before TKA and (**d**) after TKA. Error bars span ± 1 standard deviation. An asterisk (*) indicates force produced for that muscle is significantly larger compared to that in other STS phases, within the same time point. Abbreviations: AddMag, adductor magnus; GasMed, medial gastrocnemius; GluteMax, gluteus maximus; GluteMed, gluteus medius; Semimem, semimembranosus; TibAnt, tibialis anterior; VasLat, vastus lateralis; VasMed, vastus medialis

There was no significant main effect of time (before versus after TKA) on peak involved limb muscle forces (*p* = 0.521), but there were significant main effects of muscle (*p* < 0.001) and STS phase (*p* = 0.001) on peak involved limb muscle forces. There was a significant interaction between muscle and phase (*p* < 0.001), but there were no other significant interactions (*p* ≥ 0.083) from the GLM investigating peak involved limb muscle forces. At both time points, vastus lateralis produced the largest peak force during phases 2 and 3 (phase 2 before: 1011.7 ± 348.0 N; phase 2 after: 1025.6 ± 177.5 N; phase 3 before: 1078.7 ± 285.3 N, phase 3 after: 992.0 ± 195.6 N; Fig 4). Within phase 1, the largest peak force was produced by the psoas before TKA (916.3 ± 110.5 N; Figure 4) but vastus lateralis after TKA (894.5 ± 277.9 N; Fig 4). Across phases and time points, large peak forces were also produced by the soleus, vastus medialis, and gluteus medius, which were significantly larger than the peak forces produced by all other muscles except for vastus lateralis and adductor magnus (all *p* ≤ 0.035; Fig 4). At both time points, the force produced by the psoas during phase 1 was significantly larger than that produced by the psoas during phases 2 and 3 (*p* ≤ 0.003; Fig 4); there were no other significant differences in peak muscle forces between phases within the same muscle and time point.

**Fig 5.**
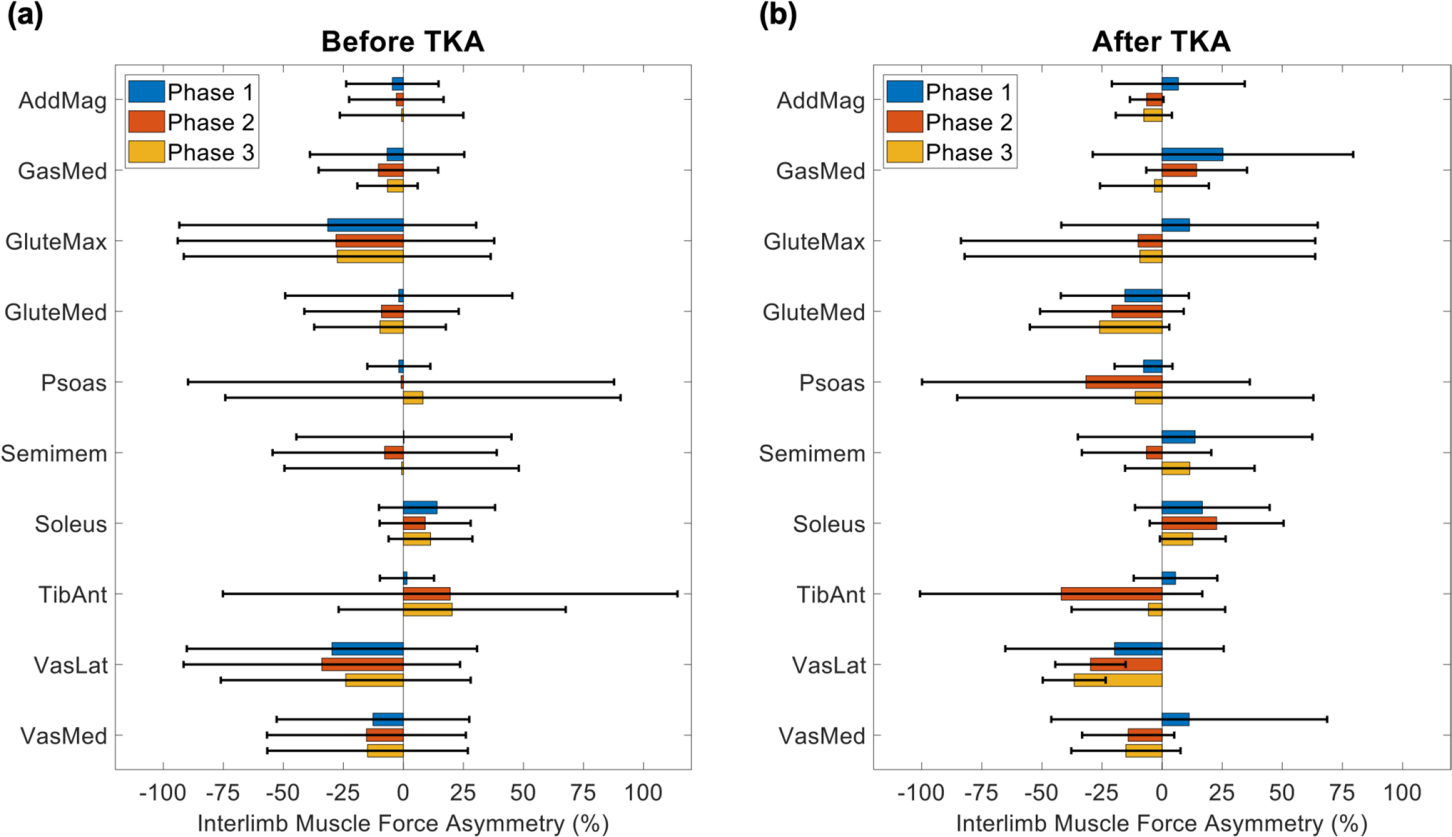
Average interlimb muscle force asymmetry across all 7 participants for each phase of the sit-to-stand (STS) cycle, (**a**) before a total knee arthroplasty (TKA) and (**b**) after TKA. Error bars span ± 1 standard deviation. All other muscles exhibited peak muscle forces of magnitude less than 400 N both before and after TKA. Abbreviations: AddMag, adductor magnus; GasMed, medial gastrocnemius; GluteMax, gluteus maximus; GluteMed, gluteus medius; Semimem, semimembranosus; TibAnt, tibialis anterior; VasLat, vastus lateralis; VasMed, vastus medialis

There were no significant main effects of time (*p* = 0.883) or phase (*p* = 0.211) on interlimb muscle force asymmetry and no significant interactions (*p* ≥ 0.126), but there was a significant main effect of muscle on interlimb muscle force asymmetry (*p* = 0.005). Before TKA, vastus lateralis demonstrated the largest interlimb muscle force asymmetry, occurring during phase 2, (− 34.0 ± 57.6%; Fig 5), indicating a smaller force from the involved limb. After TKA, tibialis anterior demonstrated the largest interlimb muscle force asymmetry, occurring during phase 2 (− 42.0 ± 58.7%; Fig 5), also indicating a smaller force from the involved limb.

### Hypothesis 3: Muscle Contributions

There was no significant main effect of time (before versus after TKA) on peak involved limb muscle contributions to vertical (*p* = 0.199) or horizontal (*p* = 0.238) COM acceleration. For both vertical and horizontal directions, there was a significant main effect of muscle (*p* < 0.001) and STS phase (*p* < 0.001) on the peak involved limb muscle contributions, with a significant interaction only between muscle and phase (*p* < 0.001).

**Fig 6.**
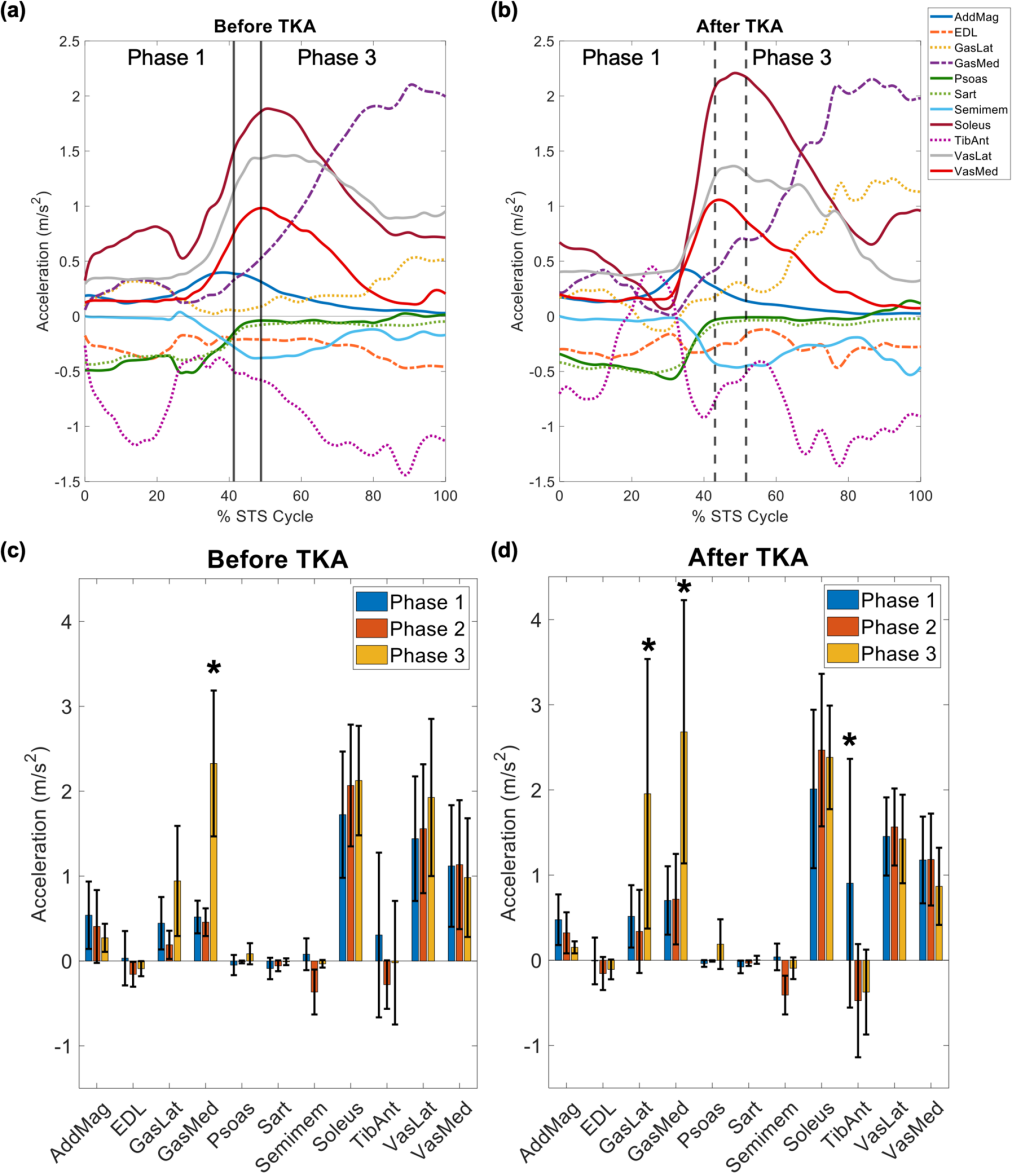
(**a**, **b**) Average muscle contributions to vertical center of mass (COM) acceleration for the involved limbs before (**a**) and after (b) total knee arthroplasty (TKA). Vertical lines denote phases of the sit-to-stand (STS) cycle. All other muscles exhibited vertical contributions of magnitude less than 0.4 m/s^2^ both before and after TKA. (**c**, **d**) Average peak muscle contributions to vertical COM acceleration across all 7 participants for each phase of the STS cycle, (**c**) before TKA and (**d**) after TKA. Error bars span ± 1 standard deviation. An asterisk (*) indicates contribution to COM acceleration for that muscle is significantly larger compared to that in other STS phases, within the same time point. Abbreviations: AddMag, adductor magnus; EDL, extensor digitorum longus; GasLat, lateral gastrocnemius; GasMed, medial gastrocnemius; Sart, sartorius; Semimem, semimembranosus; TibAnt, tibialis anterior; VasLat, vastus lateralis; VasMed, vastus medialis

At both time points, medial gastrocnemius was the largest contributor to vertical COM acceleration and reached its peak vertical contribution within phase 3 (before: 2.33 ± 0.86 m/s^2^, after: 2.68 ± 1.54 m/s^2^; Fig 6). At both time points, the vertical contribution from the medial gastrocnemius during phase 3 was significantly larger than that for phases 1 and 2 (*p* < 0.001; Fig 6). Both before and after TKA, the soleus was the largest contributor to vertical COM acceleration during phases 1 and 2 (phase 1 before: 1.72 ± 0.74 m/s^2^, phase 1 after: 2.01 ± 0.93 m/s^2^; phase 2 before: 2.07 ± 0.72 m/s^2^, phase 2 after: 2.47 ± 0.90 m/s^2^; Fig 6). At both time points, additional large contributors to vertical COM acceleration were lateral gastrocnemius and vastus medialis and lateralis, which exhibited peak contributions that were significantly larger than those exhibited by all other muscles except for medial gastrocnemius, soleus, and adductor magnus (all *p* < 0.004; Fig 6). For both time points, tibialis anterior contributed to vertical acceleration during phase 1, but contributed to vertical braking during phases 2–3; however, this difference between phases was only statistically significant after TKA (*p* ≤ 0.034; Fig 6d). Following TKA, the vertical contribution from the lateral gastrocnemius during phase 3 was significantly larger than that from the lateral gastrocnemius during phases 1 and 2 (*p* ≤ 0.004; Fig 6d); there were no other significant differences in peak vertical contributions between phases within the same muscle and time point. At both time points, tibialis anterior (phases 2–3), semimembranosus, sartorius, extensor digitorum longus (EDL), and psoas were large contributors to vertical COM braking (Fig 6, Table 2). All other muscles exhibited vertical contributions of magnitude less than 0.4 m/s^2^ both before and after TKA.

**Table 2:** Muscles with the largest contributions to horizontal and vertical center of mass (COM) acceleration (+) and braking (-), within each phase of the sit-to-stand (STS) cycle

*Direction*	Phase 1	Phase 2	Phase 3
*(+) Vertical Acceleration*^*b*^	Soleus	Soleus	Soleus
	VasLat	VasLat	VasLat
	VasMed	VasMed	VasMed
	AddMag	AddMag	MedGas
	TibAnt		LatGas
*(+) Horizontal Acceleration*^*c*^	Soleus	Soleus	Soleus
	MedGas	MedGas	MedGas
	LatGas	Semimem	Semimem
*(-) Vertical Braking*^*b*^	Sart	TibAnt	TibAnt
	Psoas	Semimem	Semimem
			EDL
*(-) Horizontal Braking*^*c*^	TibAnt	TibAnt	TibAnt
	VasLat	VasLat	VasLat
	VasMed	VasMed	VasMed
	EDL	RecFem	RecFem
			VasInt

**Fig 7.**
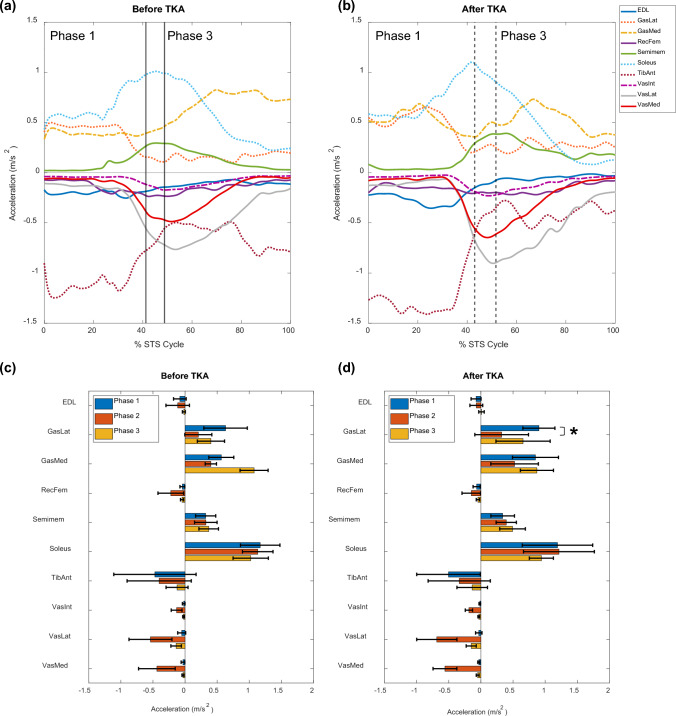
(**a**, **b**) Average muscle contributions to horizontal center of mass (COM) acceleration for the involved limbs before (**a**) and after (**b**) total knee arthroplasty (TKA). Vertical lines denote phases of the sit-to-stand (STS) cycle. All other muscles exhibited horizontal contributions of magnitude less than 0.2 m/s^2^ both before and after TKA. (**c**, **d**) Average peak muscle contributions to horizontal COM acceleration across all 7 participants for each phase of the STS cycle, (**c**) before TKA and (**d**) after TKA. Error bars span ± 1 standard deviation. An asterisk (*) indicates significant difference between phases for the same muscle and time point. Abbreviations: EDL, extensor digitorum longus; GasLat, lateral gastrocnemius; GasMed, medial gastrocnemius; RecFem, rectus femoris; Semimem, semimembranosus; TibAnt, tibialis anterior; VasInt, vastus intermedius; VasLat, vastus lateralis; VasMed, vastus medialis

Patterns are consistent between time points (before and after total knee arthroplasty (TKA)), as shown in Figs 6 and 7. Muscle abbreviations are listed below in^a^

^a^AddMag, adductor magnus; EDL, extensor digitorum longus; LatGas, lateral gastrocnemius; MedGas, medial gastrocnemius; RecFem, rectus femoris; Sart, sartorius; Semimem, semimembranosus; TibAnt, tibialis anterior; VasInt, vastus intermedius; VasLat, vastus lateralis; VasMed, vastus medialis

^b^All other muscles exhibited vertical contributions of magnitude less than 0.4 m/s^2^ both before and after TKA

^c^All other muscles exhibited horizontal contributions of magnitude less than 0.2 m/s^2^ both before and after TKA

Both before and after TKA, the soleus was the largest contributor to horizontal COM acceleration during phases 1 and 2 (phase 1 before: 1.17 ± 0.31 m/s^2^; phase 1 after: 1.19 ± 0.55 m/s^2^; phase 2 before: 1.13 ± 0.24 m/s^2^; phase 2 after: 1.21 ± 0.55 m/s^2^; Fig 7). For phase 3, the largest contributor to horizontal COM acceleration was medial gastrocnemius before TKA (1.08 ± 0.22 m/s^2^; Fig 7) but the soleus after TKA (0.94 ± 0.19 m/s^2^; Fig 7). At both time points, additional large contributors to horizontal COM acceleration were lateral gastrocnemius and semimembranosus, which exhibited contributions that were significantly larger than those exhibited by all other muscles except for medial gastrocnemius and soleus (all *p* < 0.001; Fig 7). Following TKA, the horizontal contribution from the lateral gastrocnemius during phase 1 was significantly larger than that from the lateral gastrocnemius during phase 2 (*p* = 0.02; Fig 7d); there were no other significant differences in peak vertical contributions between phases within the same muscle and time point. At both time points, the quadriceps (rectus femoris and vasti), tibialis anterior, and extensor digitorum longus (EDL) were large contributors to horizontal COM braking (Fig 7, Table 2). All other muscles exhibited horizontal contributions of magnitude less than 0.2 m/s^2^ both before and after TKA.

### Hypothesis 4: Knee Extension Strength

There was no significant difference between time points in peak involved limb knee extension strength (*p* = 0.847; before: 1.24 ± 0.38 Nm/kg; after: 1.21 ± 0.26 Nm/kg).

## Discussion

In an effort to provide insight into the persistent functional deficits often observed both before and after a primary TKA during STS, the purposes of this study were to determine whether (1) sagittal plane kinematics, (2a) muscle force production, (2b) interlimb muscle force asymmetry, (3) muscle contributions to COM acceleration, and (4) knee extension strength change following a primary TKA. Our results partially support our first hypothesis, in that there was no difference in sagittal plane peak lumbar angles, peak pelvic tilt angles, peak hip angles, peak knee angles, initial lumbar angle, initial knee angle, initial ankle angle, and initial foot placement between time points. However, there were differences in peak ankle angles, initial pelvic tilt angle, and initial hip angle between time points. Our results do not support our second and third hypotheses, where we demonstrate that muscle function during the STS transfer, when characterized by involved limb muscle force production, interlimb muscle force asymmetry, and involved limb muscle contributions to COM acceleration, does not change within 6 months following a primary TKA. Additionally, our results do not support our final hypothesis (4), where we found no differences in knee extension strength between time points.

While there were some differences in kinematic measures between time points, kinematic strategy did not largely change following TKA. After surgery, individuals demonstrated decreased dorsiflexion at the end of phase 1 (Fig 3c), as well as less posterior pelvic tilt (Fig 3e) and increased hip flexion bilaterally (Figs 3a, S5a) at the start of the STS. However, forward lumbar flexion (Fig 3d) and foot placement (Supplementary Material: Table S5) did not change between time points. Our results for forward lumbar flexion (Fig 3d) are comparable to those demonstrated previously for the KOA population during STS (peak trunk flexion of 46.4° ± 8.7°) [11], on average. On a population basis, previous work has documented greater forward trunk lean during STS for the KOA population [11], compared to healthy controls. Greater forward trunk lean places the COM closer to the base of support, as seen in the “stabilization strategy” noted by Hughes et al. [46], who found this kinematic strategy to result in longer chair rise times, compared to a “momentum transfer” kinematic strategy where the COM is farther from the base of support and the individual must overcome a larger COM moment arm and generate a larger knee torque. Greater forward trunk lean is suggestive of a strategy to reduce the demand on the quadriceps [11], which typically show strength deficits [24, 25] and activation deficits [47] that do not improve following TKA [24, 26, 48]. Results from our final hypothesis (4) support this kinematic compensation strategy of using greater forward trunk lean, as we found no difference in knee extension strength between time points. Our findings for the lack of change in knee extension strength and our values for knee extension strength are comparable to findings presented in Bade et al. [24]. On average, our presented results for knee extension strength are smaller than that exhibited by healthy controls (2.1 ± 0.5 Nm/kg) [24], suggesting a quadriceps strength deficit demonstrated by our cohort at each time point.

Muscle force production and asymmetry did not significantly differ before and after TKA, suggesting that our sample demonstrated consistent movement strategies before and after surgery to compensate for persistent quadriceps weakness on the involved side. A lack of change in muscle force asymmetry following TKA suggests that these patients’ interlimb muscle compensation strategies did not change with surgery. However, within each time point, participants in our sample exhibited no differences between limbs for any of the kinematic measures. Thus, asymmetric muscle function may be a strategy to compensate for weakness in a manner that produces larger muscle forces in the uninvolved limb, compared to the involved limb, but that results in similar sagittal plane kinematics between limbs. While our analysis was limited to the sagittal plane, others have observed asymmetric frontal plane kinematics during STS in the KOA and TKA populations, including lateral trunk lean to offload the involved limb [11, 17], which may explain the asymmetric muscle force production observed both before and after TKA, in the absence of sagittal plane kinematic asymmetry.

When comparing muscle function between populations and studies, it is important to consider differences in musculoskeletal models. Differences in the numbers of degrees of freedom, body segments, and muscles, and differences in musculoskeletal geometry and muscle properties (including muscles’ peak isometric forces), between musculoskeletal models make it difficult to directly compare estimates for simulated muscle forces between studies [20, 49, 50]. However, we can compare relative magnitudes of muscle forces and contributions between previously published data in healthy, young adults performing STS and our results, in order to identify specific muscle force production deficits and compensations that may contribute to the persistent functional performance deficits during STS often observed following TKA. For example, high forces in the gluteus maximus and vastus lateralis are observed in healthy, young adults performing STS, and these forces are large relative to other muscle forces [22]. In contrast, we see smaller gluteus maximus forces both before and after TKA, relative to other muscle forces generated by this population (Fig 4).

Muscle force production before and after TKA contrasts that exhibited by healthy, young adults performing STS [22] but is consistent with previous work that identified muscles that can compensate for simulated muscle weakness during STS [23]. Both before and after TKA, we observed relatively large forces in the psoas (beginning of phase 1; Fig 4), gluteus medius (phases 1-3; Fig 4), and soleus (phases 2–3; Fig 4), and relatively smaller forces from the gluteus maximus (phase 2–3; Fig 4) and biceps femoris (< 400 N at both time points; Supplementary Material: Fig S7). Thus, the relative magnitudes of muscle forces observed in individuals before and after TKA contrast those of healthy, young adults performing STS [22], but are generally consistent with compensation patterns predicted by models of simulated quadriceps and gluteus maximus weakness (large psoas and gluteus medius forces; small gluteus maximus and biceps femoris forces) [23].

In addition to force production, muscle contributions to horizontal and vertical COM acceleration do not significantly change within six months following TKA, and these contributions contrast those exhibited by healthy, young adults performing STS. We observed a smaller gluteus maximus contribution to acceleration in both directions for our pre/post TKA population (horizontal: < 0.2 m/s^2^; vertical: < 0.4 m/s^2^; Supplementary Material: Figs S8–9), relative to healthy, young adults [22]. Both before and after surgery, our participants demonstrated greater braking contributions to COM acceleration, especially in the vertical direction (psoas, sartorius, semimembranosus, tibialis anterior, extensor digitorum longus; Fig 6, Table 2), compared to healthy, young adults who exhibit almost no vertical COM braking during STS [22]. Greater braking contributions, which oppose the desired up and forward direction of the STS movement, may explain the persistent functional performance deficits often observed in TKA patients, especially when combined with smaller gluteus maximus contributions to upward and forward acceleration.

A muscle’s “potential” to contribute to the acceleration of a body segment or joint, such as the COM acceleration used in this study, is quantified as the muscle’s contribution to acceleration per Newton of force [51]. Kinematic modifications alter a muscle’s potential to contribute to the dynamic motion [51]. For example, with greater anterior pelvic tilt (as observed when comparing individuals with KOA to healthy, young adults during phase 2 of STS [51]), tibialis anterior’s potential to contribute to vertical and horizontal COM braking increases [51]. Thus, a muscle’s contribution to COM acceleration can increase by increasing the muscle’s potential through kinematic modifications, increasing the muscle’s force, or both. An example of this can be observed in our results by the large tibialis anterior braking contributions to both vertical (phases 2–3; Fig 6) and horizontal (all phases; Fig 7) COM acceleration, which are not observed in healthy, young adults performing STS [22]. Similarly, smaller gluteus maximus contributions (Supplementary Material: Figs S8, 9) can be explained by smaller gluteus maximus forces (phases 2–3; Fig 5), which are not observed in healthy, young adults performing STS.

This study presents several limitations, as it is a secondary analysis of prospectively collected data. We were limited in sample size due to the quality of available EMG and marker data, however our small sample size is in line with other simulation-based studies [22, 39]. The use of one representative chair rise limits the repeatability and generalizability of our results. No EMG data were available for the gluteal muscles or tibialis anterior, which may limit estimations of muscle forces computed by EMG-constrained CMC. The cost function used during CMC minimizes muscle co-activation, which is typically present in the KOA and TKA populations [15, 18], particularly between the quadriceps and hamstrings muscles. However, we constrained the model’s muscle excitations to available EMG data, and we verified that our simulated muscle activations were consistent with experimental EMG (Fig 1). Additionally, while the incorporation of the two additional degrees of freedom at the knee enabled us to capture the previously established variability of the osteoarthritic knee [33, 35] and lack of constraints in the TKA knee [34], our choice of knee model is limited in that it does not model passive structures (ligaments or, in the case of the TKA knee, the implant geometry). However, we estimated this resistance from unmodeled passive structures in the simulation by increasing the strength of the reserve torque actuators at the new degrees of freedom in the knee (Supplementary Material: Table S1), using previous findings from instrumented knee implants [52] as a starting point for these reserve torque strengths. Additionally, we verified the results of our simulations by (1) matching the experimental kinematics (RMS < 3.5 cm), (2) matching the experimental EMG, and (3) ensuring that the muscle forces when multiplied by their respective moment arms and then summed about a joint matched the inverse dynamics joint torques (for our major muscles and joint torques of interest in the sagittal plane).

We demonstrated through musculoskeletal simulations that participants demonstrated consistent movement strategies (quantified by kinematics and muscle function) before and six months after TKA, as an accommodation for persistent quadriceps weakness. Moreover, these individuals before and after TKA exhibited deficits and compensations in muscle force production, including large psoas, gluteus medius, and soleus forces and small gluteus maximus and biceps femoris forces. Additionally, before and after TKA, individuals demonstrated large braking contributions, particularly in the vertical direction, and small gluteus maximus contributions to COM acceleration. These larger braking contributions, combined with smaller forward and upward contributions to COM acceleration, may explain the functional performance limitations typically observed in individuals before and after TKA. Future work should investigate whether this lack of change in muscle function carries over to other activities of daily living and should further explore the relationship between changes in muscle strength and kinematics on TKA patients’ ability to perform these activities of daily living.

## Supplementary Information

Below is the link to the electronic supplementary material.

Supplementary file1 (PDF 2022 KB)

## References

[1] Bohannon, R. W. Daily sit-to-stands performed by adults: a systematic review. *J Phys Ther Sci.* 27:939–942, 2015. 10.1589/jpts.27.939. 25931764 10.1589/jpts.27.939 PMC4395748

[2] Lindemann, U., R. Muche, M. Stuber, W. Zijlstra, K. Hauer, and C. Becker. Coordination of strength exertion during the chair-rise movement in very old people. *J Gerontol Ser A.* 62:636–640, 2007. 10.1093/gerona/62.6.636. 10.1093/gerona/62.6.636 17595420

[3] Hughes, M. A., B. S. Myers, and M. L. Schenkman. The role of strength in rising from a chair in the functionally impaired elderly. *J Biomech.* 29:1509–1513, 1996. 10.1016/S0021-9290(96)80001-7. 8945648

[4] Christiansen, C. L., and J. E. Stevens-Lapsley. Weight-bearing asymmetry in relation to measures of impairment and functional mobility for people with knee osteoarthritis. *Arch Phys Med Rehabil.* 91:1524–1528, 2010. 10.1016/j.apmr.2010.07.009. 20875509 10.1016/j.apmr.2010.07.009 PMC2948025

[5] Su, F. C., K. A. Lai, and W. H. Hong. Rising from chair after total knee arthroplasty. *Clin Biomech.* 13:176–181, 1998. 10.1016/S0268-0033(97)00039-9. 10.1016/s0268-0033(97)00039-9 11415785

[6] Jones, C. A., D. C. Voaklander, D. W. Johnston, and M. E. Suarez-Almazor. Health related quality of life outcomes after total hip and knee arthroplasties in a community based population. *J Rheumatol.* 27:1745–1752, 2000. 10914862

[7] Stevens-Lapsley, J. E., M. L. Schenkman, and M. R. Dayton. Comparison of self-reported knee injury and osteoarthritis outcome score to performance measures in patients after total knee arthroplasty. *PM&R.* 3:541–549, 2011. 10.1016/j.pmrj.2011.03.002. 21665167 10.1016/j.pmrj.2011.03.002

[8] Parvizi, J., R. M. Nunley, K. R. Berend, A. V. Lombardi, E. L. Ruh, J. C. Clohisy, et al. High level of residual symptoms in young patients after total knee arthroplasty. *Clin Orthop.* 472:133–137, 2014. 10.1007/s11999-013-3229-7. 24061845 10.1007/s11999-013-3229-7 PMC3889453

[9] Boonstra, M. C., M. C. De Waal Malefijt, and N. Verdonschot. How to quantify knee function after total knee arthroplasty? *The Knee.* 15:390–395, 2008. 10.1016/j.knee.2008.05.006. 18620863 10.1016/j.knee.2008.05.006

[10] Sonoo, M., H. Iijima, and N. Kanemura. Altered sagittal plane kinematics and kinetics during sit-to-stand in individuals with knee osteoarthritis: a systematic review and meta-analysis. *J Biomech.* 96:109331, 2019. 10.1016/j.jbiomech.2019.109331. 31610881 10.1016/j.jbiomech.2019.109331

[11] Turcot, K., S. Armand, D. Fritschy, P. Hoffmeyer, and D. Suvà. Sit-to-stand alterations in advanced knee osteoarthritis. *Gait Posture.* 36:68–72, 2012. 10.1016/j.gaitpost.2012.01.005. 22326239 10.1016/j.gaitpost.2012.01.005

[12] Wang, J., S. F. Siddicky, T. E. Oliver, M. P. Dohm, C. L. Barnes, and E. M. Mannen. Biomechanical changes following knee arthroplasty during sit-to-stand transfers: systematic review. *J Arthroplasty.* 34:2494–2501, 2019. 10.1016/j.arth.2019.05.028. 31186182 10.1016/j.arth.2019.05.028

[13] Farquhar, S. J., D. S. Reisman, and L. Snyder-Mackler. Persistence of altered movement patterns during a sit-to-stand task 1 year following unilateral total knee arthroplasty. *Phys Ther.* 88:567–579, 2008. 10.2522/ptj.20070045. 18292217 10.2522/ptj.20070045

[14] Naili, J. E., E. W. Broström, E. M. Gutierrez-Farewik, and M. H. Schwartz. The centre of mass trajectory is a sensitive and responsive measure of functional compensations in individuals with knee osteoarthritis performing the five times sit-to-stand test. *Gait Posture.* 62:140–145, 2018. 10.1016/j.gaitpost.2018.03.016. 29549868 10.1016/j.gaitpost.2018.03.016

[15] Bouchouras, G., G. Patsika, V. Hatzitaki, and E. Kellis. Kinematics and knee muscle activation during sit-to-stand movement in women with knee osteoarthritis. *Clin Biomech.* 30:599–607, 2015. 10.1016/j.clinbiomech.2015.03.025. 10.1016/j.clinbiomech.2015.03.025 25846323

[16] Jevsevar, D. S., P. O. Riley, W. A. Hodge, and D. E. Krebs. Knee kinematics and kinetics during locomotor activities of daily living in subjects with knee arthroplasty and in healthy control subjects. *Phys Ther.* 73:229–239, 1993. 10.1093/ptj/73.4.229. 8456142 10.1093/ptj/73.4.229

[17] Mizner, R. L., and L. Snyder-Mackler. Altered loading during walking and sit-to-stand is affected by quadriceps weakness after total knee arthroplasty. *J Orthop Res.* 23:1083–1090, 2005. 10.1016/j.orthres.2005.01.021. 16140191 10.1016/j.orthres.2005.01.021

[18] Davidson, B. S., D. L. Judd, A. C. Thomas, R. L. Mizner, D. G. Eckhoff, and J. E. Stevens-Lapsley. Muscle activation and coactivation during five-time-sit-to-stand movement in patients undergoing total knee arthroplasty. *J Electromyogr Kinesiol.* 23:1485–1493, 2013. 10.1016/j.jelekin.2013.06.008. 23953763 10.1016/j.jelekin.2013.06.008

[19] Delp, S. L., F. C. Anderson, A. S. Arnold, P. Loan, A. Habib, C. T. John, et al. OpenSim: open-source software to create and analyze dynamic simulations of movement. *IEEE Trans Biomed Eng.* 54:1940–1950, 2007. 10.1109/TBME.2007.901024. 18018689 10.1109/TBME.2007.901024

[20] Hicks, J. L., T. K. Uchida, A. Seth, A. Rajagopal, and S. L. Delp. Is my model good enough? Best practices for verification and validation of musculoskeletal models and simulations of movement. *J Biomech Eng.* 2015. 10.1115/1.4029304. 25474098 10.1115/1.4029304 PMC4321112

[21] Zajac, F. E., and M. E. Gordon. Determining muscle’s force and action in multi-articular movement. *Exerc Sport Sci Rev.* 17:187–230, 1989. 2676547

[22] Caruthers, E. J., J. A. Thompson, A. M. W. Chaudhari, L. C. Schmitt, T. M. Best, K. R. Saul, et al. Muscle forces and their contributions to vertical and horizontal acceleration of the center of mass during sit-to-stand transfer in young Healthy Adults. *J Appl Biomech.* 32:487–503, 2016. 10.1123/jab.2015-0291. 27341083 10.1123/jab.2015-0291

[23] Caruthers, E. J., G. Schneider, L. C. Schmitt, A. M. W. Chaudhari, and R. A. Siston. What are the effects of simulated muscle weakness on the sit-to-stand transfer? *Comput Methods Biomech Biomed Engin.* 23:765–772, 2020. 10.1080/10255842.2020.1764544. 32469249 10.1080/10255842.2020.1764544

[24] Bade, M. J., W. M. Kohrt, and J. E. Stevens-Lapsley. Outcomes before and after total knee arthroplasty compared to healthy adults. *J Orthop Sports Phys Ther.* 40:559–567, 2010. 10.2519/jospt.2010.3317. 20710093 10.2519/jospt.2010.3317 PMC3164265

[25] Messier, S. P., R. F. Loeser, J. L. Hoover, E. L. Semble, and C. M. Wise. Osteoarthritis of the knee: effects on gait, strength, and flexibility. *Arch Phys Med Rehabil.* 73:29–36, 1992. 10.5555/uri:pii:000399939290222I. 1729969

[26] Walsh, M., L. J. Woodhouse, S. G. Thomas, and E. Finch. Physical impairments and functional limitations: a comparison of individuals 1 year after total knee arthroplasty with control subjects. *Phys Ther.* 78:248–258, 1998. 10.1093/ptj/78.3.248. 9520970 10.1093/ptj/78.3.248

[27] Freisinger, G. M., E. E. Hutter, J. Lewis, J. F. Granger, A. H. Glassman, M. D. Beal, et al. Relationships between varus–valgus laxity of the severely osteoarthritic knee and gait, instability, clinical performance, and function. *J Orthop Res.* 35:1644–1652, 2017. 10.1002/jor.23447. 27664972 10.1002/jor.23447 PMC5678997

[28] Chaudhari, A. M. W., L. C. Schmitt, G. M. Freisinger, J. M. Lewis, E. E. Hutter, X. Pan, et al. Perceived instability is associated with strength and pain, not frontal knee laxity, in patients with advanced knee osteoarthritis. *J Orthop Sports Phys Ther.* 49:513–517, 2019. 10.2519/jospt.2019.8619. 31213160 10.2519/jospt.2019.8619 PMC7057762

[29] Koehn, R. R., S. A. Roelker, X. Pan, L. C. Schmitt, A. M. W. Chaudhari, and R. A. Siston. Is modular control related to functional outcomes in individuals with knee osteoarthritis and following total knee arthroplasty? *PLOS ONE.* 17:e0267340, 2022. 10.1371/journal.pone.0267340. 35452480 10.1371/journal.pone.0267340 PMC9032423

[30] Jamison, S. T., X. Pan, and A. M. W. Chaudhari. Knee moments during run-to-cut maneuvers are associated with lateral trunk positioning. *J Biomech.* 45:1881–1885, 2012. 10.1016/j.jbiomech.2012.05.031. 22704608 10.1016/j.jbiomech.2012.05.031

[31] Schenkman, M., R. A. Berger, P. O. Riley, R. W. Mann, and W. A. Hodge. Whole-body movements during rising to standing from sitting. *Phys Ther.* 70:638–648, 1990. 10.1093/ptj/70.10.638. 2217543 10.1093/ptj/70.10.638

[32] Bosch, W., A. Esrafilian, P. Vartiainen, J. Arokoski, R. K. Korhonen, and L. Stenroth. Alterations in the functional knee alignment are not an effective strategy to modify the mediolateral distribution of knee forces during closed kinetic chain exercises. *J Appl Biomech.* 38:424–433, 2022. 10.1123/jab.2021-0310. 36395764 10.1123/jab.2021-0310

[33] Kumar, D., K. T. Manal, and K. S. Rudolph. Knee joint loading during gait in healthy controls and individuals with knee osteoarthritis. *Osteoarthr Cartil OARS Osteoarthr Res Soc.* 21:298–305, 2013. 10.1016/j.joca.2012.11.008. 10.1016/j.joca.2012.11.008 PMC3804122 23182814

[34] Watanabe, T., H. Koga, H. Katagiri, K. Otabe, Y. Nakagawa, T. Muneta, et al. Coronal and sagittal laxity affects clinical outcomes in posterior-stabilized total knee arthroplasty: assessment of well-functioning knees. *Knee Surg Sports Traumatol Arthrosc.* 28:1400–1409, 2020. 10.1007/s00167-019-05500-8. 30980120 10.1007/s00167-019-05500-8

[35] Siston, R. A., N. J. Giori, S. B. Goodman, and S. L. Delp. Intraoperative passive kinematics of osteoarthritic knees before and after total knee arthroplasty. *J Orthop Res.* 24:1607–1614, 2006. 10.1002/jor.20163. 16770795 10.1002/jor.20163

[36] Uhlrich, S. D., R. W. Jackson, A. Seth, J. A. Kolesar, and S. L. Delp. Muscle coordination retraining inspired by musculoskeletal simulations reduces knee contact force. *Sci Rep.* 12:9842, 2022. 10.1038/s41598-022-13386-9. 35798755 10.1038/s41598-022-13386-9 PMC9262899

[37] Thelen, D. G., F. C. Anderson, and S. L. Delp. Generating dynamic simulations of movement using computed muscle control. *J Biomech.* 36:321–328, 2003. 10.1016/S0021-9290(02)00432-3. 12594980 10.1016/s0021-9290(02)00432-3

[38] Thelen, D. G., and F. C. Anderson. Using computed muscle control to generate forward dynamic simulations of human walking from experimental data. *J Biomech.* 39:1107–1115, 2006. 10.1016/j.jbiomech.2005.02.010. 16023125 10.1016/j.jbiomech.2005.02.010

[39] Roelker, S. A., P. DeVita, J. D. Willson, and R. R. Neptune. Differences in muscle demand and joint contact forces between running and skipping. *J Appl Biomech.* 38:382–390, 2022. 10.1123/jab.2022-0011. 36265840 10.1123/jab.2022-0011

[40] Gaffney, B. M., M. D. Harris, B. S. Davidson, J. E. Stevens-Lapsley, C. L. Christiansen, and K. B. Shelburne. Multi-joint compensatory effects of unilateral total knee arthroplasty during high-demand tasks. *Ann Biomed Eng.* 44:2529–2541, 2016. 10.1007/s10439-015-1524-z. 26666227 10.1007/s10439-015-1524-z PMC4907879

[41] Roelker, S. A., E. J. Caruthers, R. K. Hall, N. C. Pelz, A. M. W. Chaudhari, and R. A. Siston. Effects of optimization technique on simulated muscle activations and forces. *J Appl Biomech.* 36:259–278, 2020. 10.1123/jab.2018-0332. 32663800 10.1123/jab.2018-0332

[42] Anderson, F. C., and M. G. Pandy. Individual muscle contributions to support in normal walking. *Gait Posture.* 17:159–169, 2003. 10.1016/S0966-6362(02)00073-5. 12633777 10.1016/s0966-6362(02)00073-5

[43] Hamner, S. R., A. Seth, and S. L. Delp. Muscle contributions to propulsion and support during running. *J Biomech.* 43:2709–2716, 2010. 10.1016/j.jbiomech.2010.06.025. 20691972 10.1016/j.jbiomech.2010.06.025 PMC2973845

[44] Liu, M. Q., F. C. Anderson, M. G. Pandy, and S. L. Delp. Muscles that support the body also modulate forward progression during walking. *J Biomech.* 39:2623–2630, 2006. 10.1016/j.jbiomech.2005.08.017. 16216251 10.1016/j.jbiomech.2005.08.017

[45] Hamner, S. R., A. Seth, K. M. Steele, and S. L. Delp. A rolling constraint reproduces ground reaction forces and moments in dynamic simulations of walking, running, and crouch gait. *J Biomech.* 46:1772–1776, 2013. 10.1016/j.jbiomech.2013.03.030. 23702045 10.1016/j.jbiomech.2013.03.030 PMC3993009

[46] Hughes, M. A., D. K. Weiner, M. L. Schenkman, R. M. Long, and S. A. Studenski. Chair rise strategies in the elderly. *Clin Biomech.* 9:187–192, 1994. 10.1016/0268-0033(94)90020-5. 10.1016/0268-0033(94)90020-5 23916180

[47] Pietrosimone, B. G., J. Hertel, C. D. Ingersoll, J. M. Hart, and S. A. Saliba. Voluntary quadriceps activation deficits in patients with tibiofemoral osteoarthritis: a meta-analysis. *PM&R.* 3:153–162, 2011. 10.1016/j.pmrj.2010.07.485. 21333954 10.1016/j.pmrj.2010.07.485

[48] Hubley-Kozey, C. L., G. L. Hatfield, J. L. A. Wilson, and M. J. Dunbar. Alterations in neuromuscular patterns between pre and one-year post-total knee arthroplasty. *Clin Biomech.* 25:995–1002, 2010. 10.1016/j.clinbiomech.2010.07.008. 10.1016/j.clinbiomech.2010.07.008 20728970

[49] Roelker, S. A., E. J. Caruthers, R. K. Baker, N. C. Pelz, A. M. W. Chaudhari, and R. A. Siston. Interpreting musculoskeletal models and dynamic simulations: causes and effects of differences between models. *Ann Biomed Eng.* 45:2635–2647, 2017. 10.1007/s10439-017-1894-5. 28779473 10.1007/s10439-017-1894-5

[50] Wagner, D. W., V. Stepanyan, J. M. Shippen, M. S. DeMers, R. S. Gibbons, B. J. Andrews, et al. Consistency among musculoskeletal models: caveat utilitor. *Ann Biomed Eng.* 41:1787–1799, 2013. 10.1007/s10439-013-0843-1. 23775441 10.1007/s10439-013-0843-1

[51] Roelker, S. A., L. C. Schmitt, A. M. W. Chaudhari, and R. A. Siston. Discover your potential: the influence of kinematics on a muscle’s ability to contribute to the sit-to-stand transfer. *PLOS ONE.* 17:e0264080, 2022. 10.1371/journal.pone.0264080. 35239690 10.1371/journal.pone.0264080 PMC8893693

[52] Kutzner, I., B. Heinlein, F. Graichen, A. Bender, A. Rohlmann, A. Halder, et al. Loading of the knee joint during activities of daily living measured *in vivo* in five subjects. *J Biomech.* 43:2164–2173, 2010. 10.1016/j.jbiomech.2010.03.046. 20537336 10.1016/j.jbiomech.2010.03.046

