# Supplementary material for: Do Kinematics or Muscle Function During Sit-to-Stand Change Following a Primary Total Knee Arthroplasty?
Source: Ann Biomed Eng. 2025 Jul 21;53(10):2474–88. doi: 10.1007/s10439-025-03782-3 (PMC12457571; doi:10.1007/s10439-025-03782-3)
Supplement: Supplementary file 1 — Supplementary file1 (PDF 2022 KB) [file 10439_2025_3782_MOESM1_ESM.pdf]

Supplementary Material

Sit-to-Stand Phases

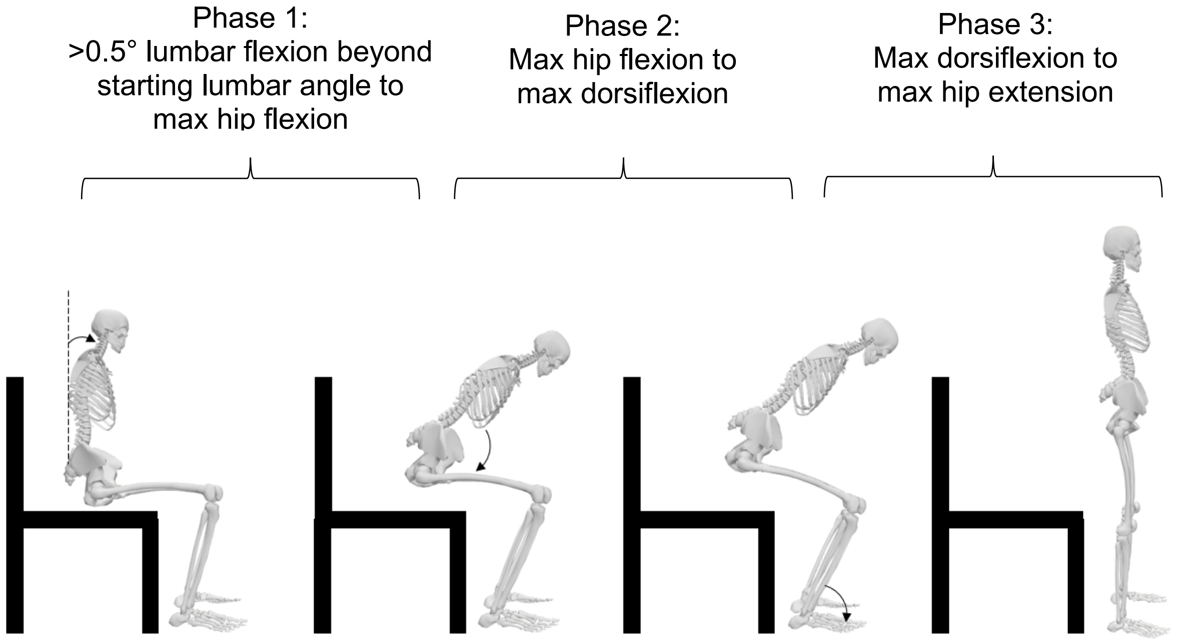

Figure S1. Definitions for each phase of the sit-to-stand cycle, as established by Schenkman et al. [1,2]. Each phase is determined by distinct kinematic events.

Model Adjustments

To better model the knee joint laxity demonstrated by the knee osteoarthritis (KOA) and total knee arthroplasty (TKA) populations [3,4], we modified the Bosch et al. model to include two additional degrees of freedom (DOFs) at the knee: knee adduction/abduction and knee internal/external rotation. Each of these DOFs was limited to a range of -10 to 10 degrees [5,6].

To improve estimates of simulated muscle activations during computed muscle control (CMC), we increased the strengths of reserve torque actuators for the hip and knee adduction/abduction and internal/external rotation DOFs. These reserve torque actuators represent unmodeled passive structures, such as resistance from an implant, soft tissue or ligamentous structures. Table S1 shows the values for the strengths of the reserve torque actuators.

Table S1. Reserve torque actuator strengths for the hip and knee adduction/abduction and internal/external rotation degrees of freedom.

Degree of Freedom	Strength (Nm)
Hip adduction/abduction	65
Hip internal/external rotation	30
Knee adduction/abduction	60
Knee internal/external rotation	20

Additionally, the origin and insertion points of the hip abductor muscles were modified to better match experimentally determined moment arms and to improve estimates of hip flexor muscle activations, as done in Uhlrich et al. [7].

Finally, we also modified the passive muscle force properties of each muscle by calibrating each muscle's passive force-length curve, such that passive joint moments generated by the model's muscles more closely matched experimental data [7,8]. In our calibration, we increased the calibration range beyond that used by Uhlrich et al. in order to better capture the range of motion typically used during sit-to-stand (STS) transfers, without extrapolating beyond the experimental passive moment data [8]. Silder et al.'s experimental limits [8] for the sagittal plane hip, knee, and ankle angles were used as our calibration range and are listed below in Table S2, and a comparison of the original and updated models' passive joint moments is shown for a set of experimental joint angles is shown in Figure S2. The final, updated passive muscle force properties are listed in Table S3. A comparison simulated muscle activations for the original Bosch model, our final model, and (when available) EMG data is shown in Figure S3 for one representative participant.

Table S2. Range of experimental passive moment data used in calibration of passive muscle force properties.

Joint	Calibration Limit (°)
Hip	-15° extension<br>80° flexion
Knee	0° extension<br>120° flexion
Ankle	-30° plantarflexion<br>20° dorsiflexion

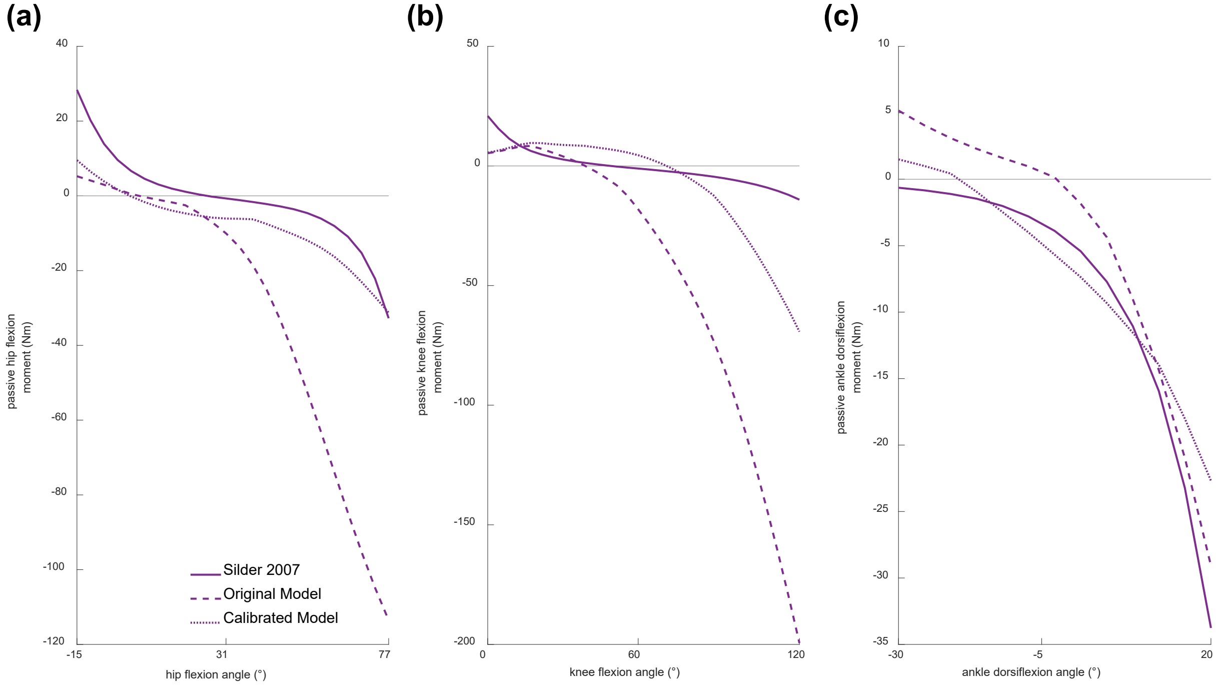

Figure S2. Comparison of original and calibrated models' passive joint moments, along with experimental passive joint moments for 3 sets of experimental joint angle ranges. For (a), hip flexion varied from -15° extension to 77° flexion, while knee and ankle were fixed at 15° flexion and 0° dorsiflexion, respectively. For (b), knee flexion varied from 0° to 120° flexion, while hip and ankle angles were fixed at 0° flexion and 20° dorsiflexion, respectively. For (c), ankle angle varied from -30° plantarflexion to 20° dorsiflexion, while hip and knee angles were both fixed at 0°. For each plot (a-c), the calibrated model's passive joint moments more closely match experimental passive joint moments.

Table S3. Calibrated passive muscle force curve parameters ("strain_at_zero_force" and "strain_at_one_norm_force") for the musculoskeletal model.

Muscle Name		Muscle Name Abbreviation	$\frac{l^m}{l_0^m}$ at $F^m = 0$ strain_at_zero_force	$\frac{l^m}{l_0^m}$ at $F^m = F_0^m$ strain_at_one_norm_force
**Default Value**			1.00	1.70
**Hip Flexors**	Psoas	psoas	1.06	1.66
	Iliacus	iliacus	0.91	1.50
	Adductor Longus	addlong	0.84	1.50
	Tensor Fascia Latae	tfl	1.20	1.71
**Gluteus Maximus**	Gluteus Maximus (Superior)	glmax1	1.11	1.70
	Gluteus Maximus (Middle)	glmax2	1.15	1.70
	Gluteus Maximus (Inferior)	glmax3	1.20	1.72
**Hamstrings**	Biceps Femoris Long Head	bflh	1.20	1.90
	Biceps Femoris Short Head	bfsh	1.06	1.70
	Semimembranosus	semimem	1.20	1.90
	Semitendinosus	semiten	1.20	1.90
**Quadriceps**	Vastus Medialis	vasmed	1.20	1.90
	Vastus Lateralis	vaslat	1.20	1.90
	Vastus Intermedius	vasint	1.20	1.90
	Rectus Femoris	recfem	1.20	1.90
**Ankle Dorsiflexors**	Tibialis Anterior	tibant	1.17	1.71
**Ankle Plantarflexors**	Gastrocnemius Medialis	gasmed	0.8	1.85
	Gastrocnemius Lateralis	gaslat	1.19	1.71
	Soleus	soleus	1.13	1.73
	Tibialis Posterior	tibpost	1.02	1.70
**Other**	Adductor Brevis	addbrev	0.94	1.65
	Adductor Magnus (Distal)	addmagDist	1.08	1.70
	Adductor Magnus (Ischial)	addmagIsch	1.20	1.71
	Adductor Magnus (Middle)	addmagMid	1.00	1.70
	Adductor Magnus (Proximal)	addmagProx	1.00	1.70
	Extensor Digitorum Longus	edl	1.10	1.71
	Extensor Hallucis Longus	ehl	1.07	1.72
	Flexor Digitorum Longus	fdl	1.00	1.70
	Flexor Hallucis Longus	fhl	1.00	1.70
	Gluteus Medius (Anterior)	glmed1	0.80	1.50
	Gluteus Medius (Middle)	glmed2	0.80	1.50
	Gluteus Medius (Posterior)	glmed3	0.95	1.90
	Gluteus Minimus (Anterior)	glmin1	1.12	1.70
	Gluteus Minimus (Middle)	glmin2	1.03	1.77
	Gluteus Minimus (Posterior)	glmin3	1.00	1.70
	Gracilis	grac	1.20	1.72
	Peroneus Brevis	perbrev	1.01	1.70
	Peroneus Longus	perlong	1.03	1.70
	Piriformis	piri	1.20	1.76
	Sartorius	sart	0.80	1.50

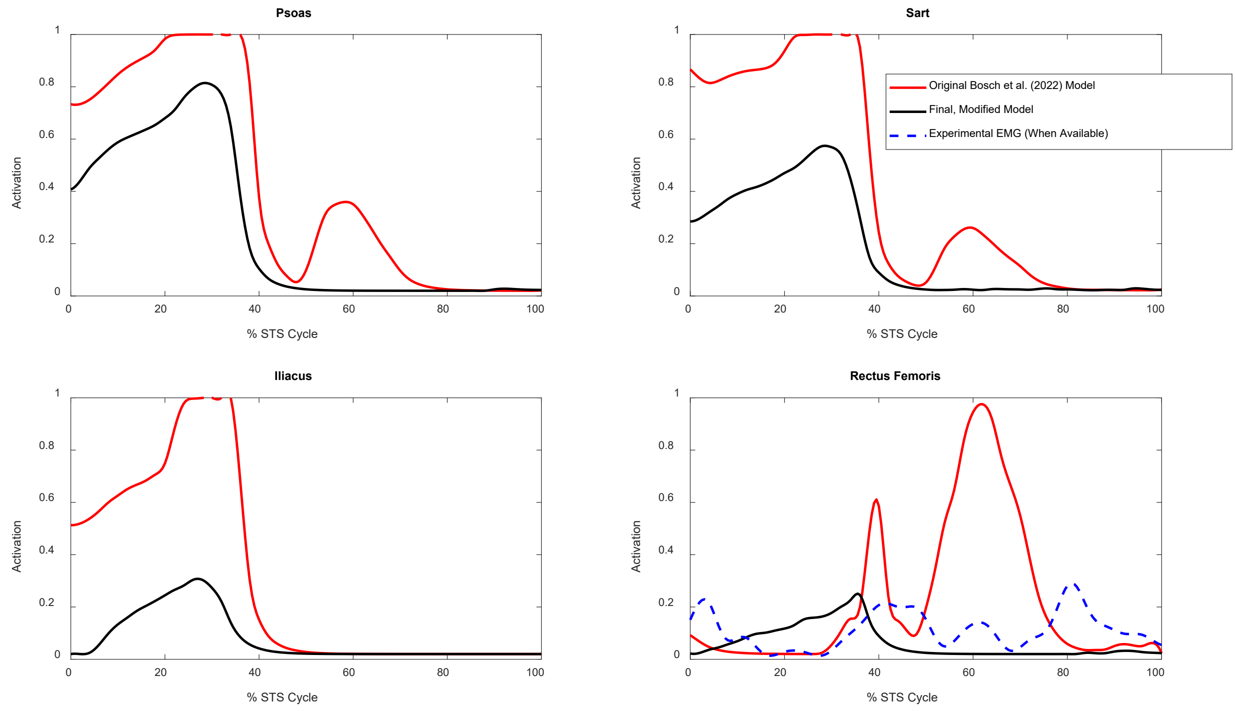

Figure S3. Simulated muscle activations (from computed muscle control) for a few hip flexor muscles (psoas, sartorius, iliacus, and rectus femoris) using the original Bosch model and modified model (with increased hip and knee reserve torque actuators, modified hip abductor paths, and calibrated passive muscle force properties) for a representative participant after total knee arthroplasty (TKA). The updated model more closely matches the magnitude of experimental electromyography (EMG) when available [9] and produces more physiologically-reasonable muscle activations.

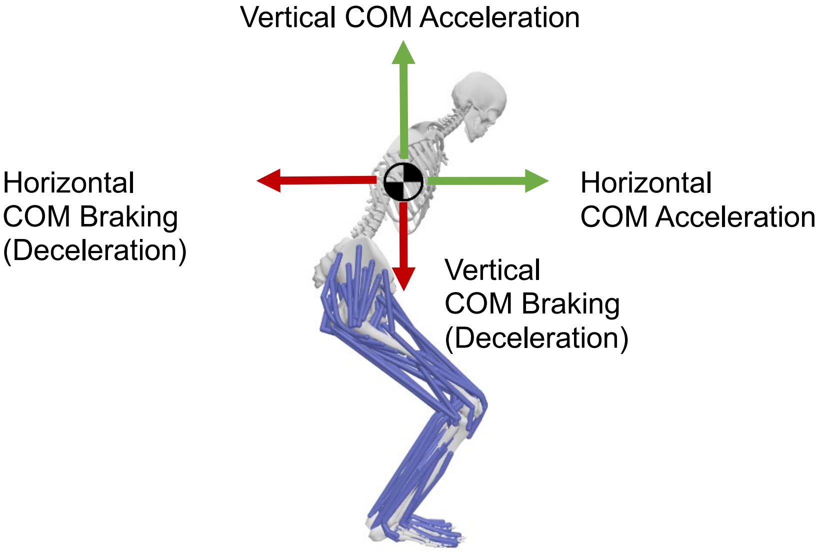

Figure S4. Definitions for induced acceleration terminology. As done in [1], vertical and horizontal accelerations represent the acceleration of the center of mass (COM) in the upward and forward directions, respectively. Vertical and horizontal braking represent the deceleration (braking) of the COM in the upward and forward directions.

Overview of General Linear Models

Table S4. Summary of the general linear models (GLM) used for each response variable during sit-to-stand (STS) in patients before and after total knee arthroplasty (TKA). For all GLMs, participant was treated as a random factor.

Response Variables (& Corresponding Hypotheses)	Random Factor	Fixed Factors
Initial sagittal plane hip, knee, or ankle angle, at 0% of STS (1)	Participant	• Timepoint (before or after TKA)<br>• Limb (involved or uninvolved)
Peak sagittal plane hip, knee, or ankle angle, per phase of STS (1)	Participant	• Timepoint (before or after TKA)<br>• Limb (involved or uninvolved)<br>• Phase of STS (1, 2, or 3)
Peak sagittal plane lumbar or pelvic tilt angle, per phase of STS (1)	Participant	• Timepoint (before or after TKA)<br>• Phase of STS (1, 2, or 3)
Peak per STS phase of: muscle forces (2a), interlimb muscle force asymmetry (2b), muscle contributions to horizontal center of mass acceleration (3), or muscle contributions to vertical center of mass acceleration (3)	Participant	• Timepoint (before or after TKA)<br>• Muscle<br>• Phase of STS (1, 2, or 3)

Initial Foot Placement Calculation

We defined initial anteroposterior foot placement as the distance, in the anterior direction, between the pelvis center of mass (COM) and involved limb's heel marker (RHEE for right-involved; LHEE for left-involved) at the start of the STS cycle (0% of STS). Equation S1 below depicts this calculation for foot placement (X_{dist}), and Figure S5 below shows this distance for a right-involved subject.

$$X_{dist} = RHEE_x - (Pelvis\ COM)_x \qquad \text{Equation S1}$$

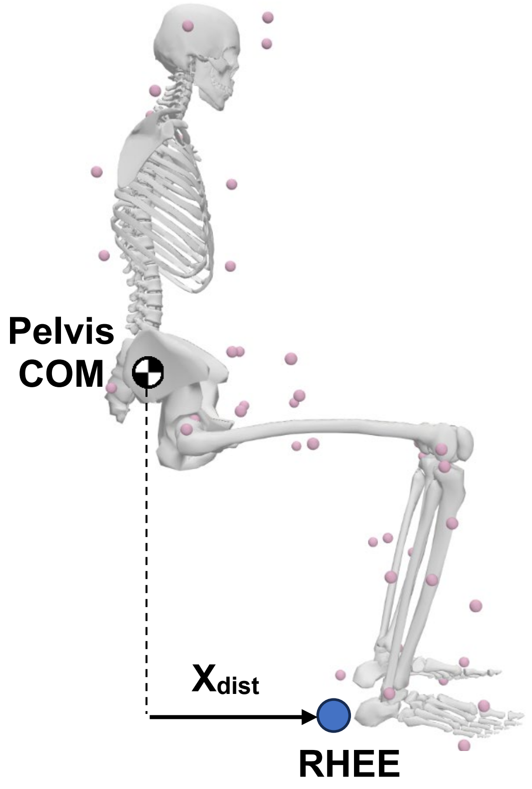

Figure S5. Initial anteroposterior foot placement (X_{dist}) was defined by the anterior distance from the pelvis center of mass (COM) to the involved limb's heel marker (e.g. RHEE for a right-involved subject).

Results for the initial foot placement are depicted below in Table S5 for each time point.

Table S5. Mean ± standard deviation of initial foot placement for each time point.

Before TKA	After TKA
0.27 ± 0.03 m	0.25 ± 0.05 m

Uninvolved Limb Kinematics

The uninvolved limb's hip, knee, and ankle sagittal plane kinematics are shown below in Figure S6, indicating a significant difference between time points (before versus after TKA) only for the initial hip flexion angle (Figure S6a).

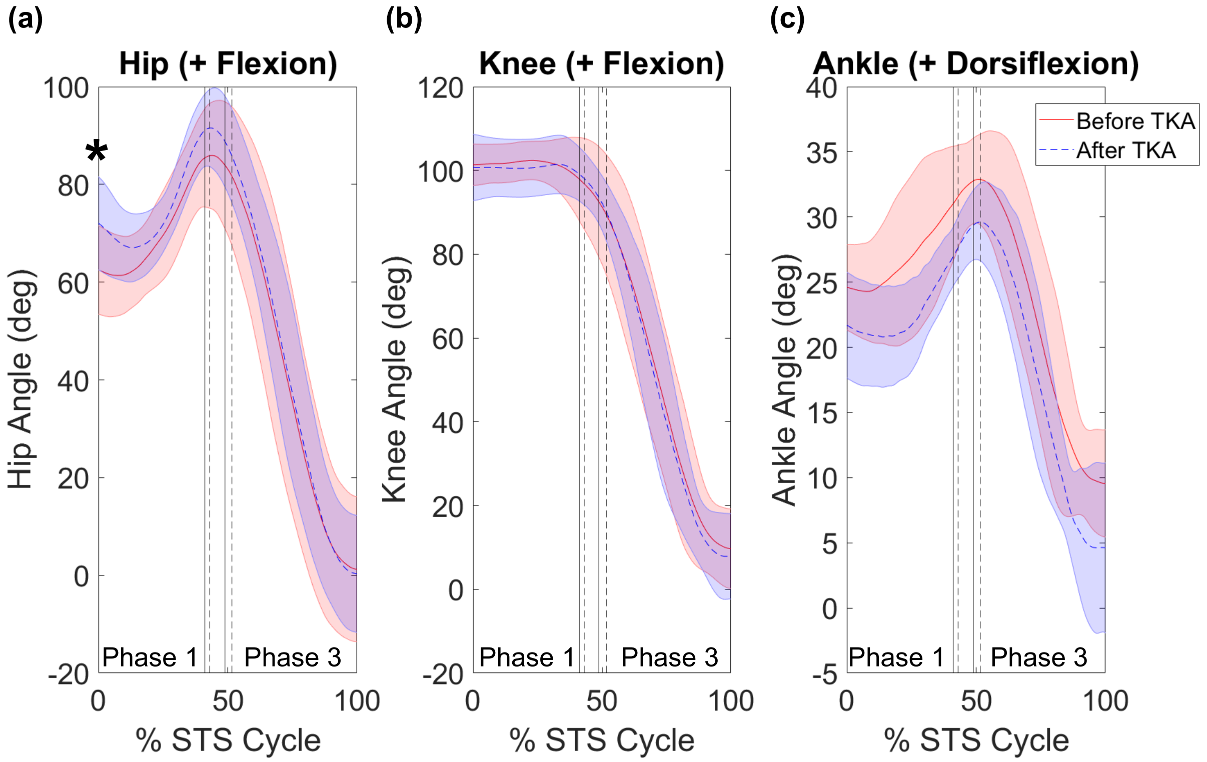

Figure S6. Mean ± standard deviation of uninvolved sagittal plane hip (a), knee (b), and ankle (c) angles before (red) and after (blue) a total knee arthroplasty (TKA) over the sit-to-stand (STS) cycle. Phases of the STS cycle are indicated by black vertical lines, with solid lines indicating before TKA and dashed indicating after TKA. In (a), * indicates significant difference in initial hip angle before and after TKA.

Involved Limb Muscle Forces

The involved limb's muscle forces at each time point (before and after TKA) are shown below in Figure S7, with the force produced by biceps femoris appended to plots presented in Figure 4. At each time point, the biceps femoris force is small, relative to the forces produced by the other muscles.

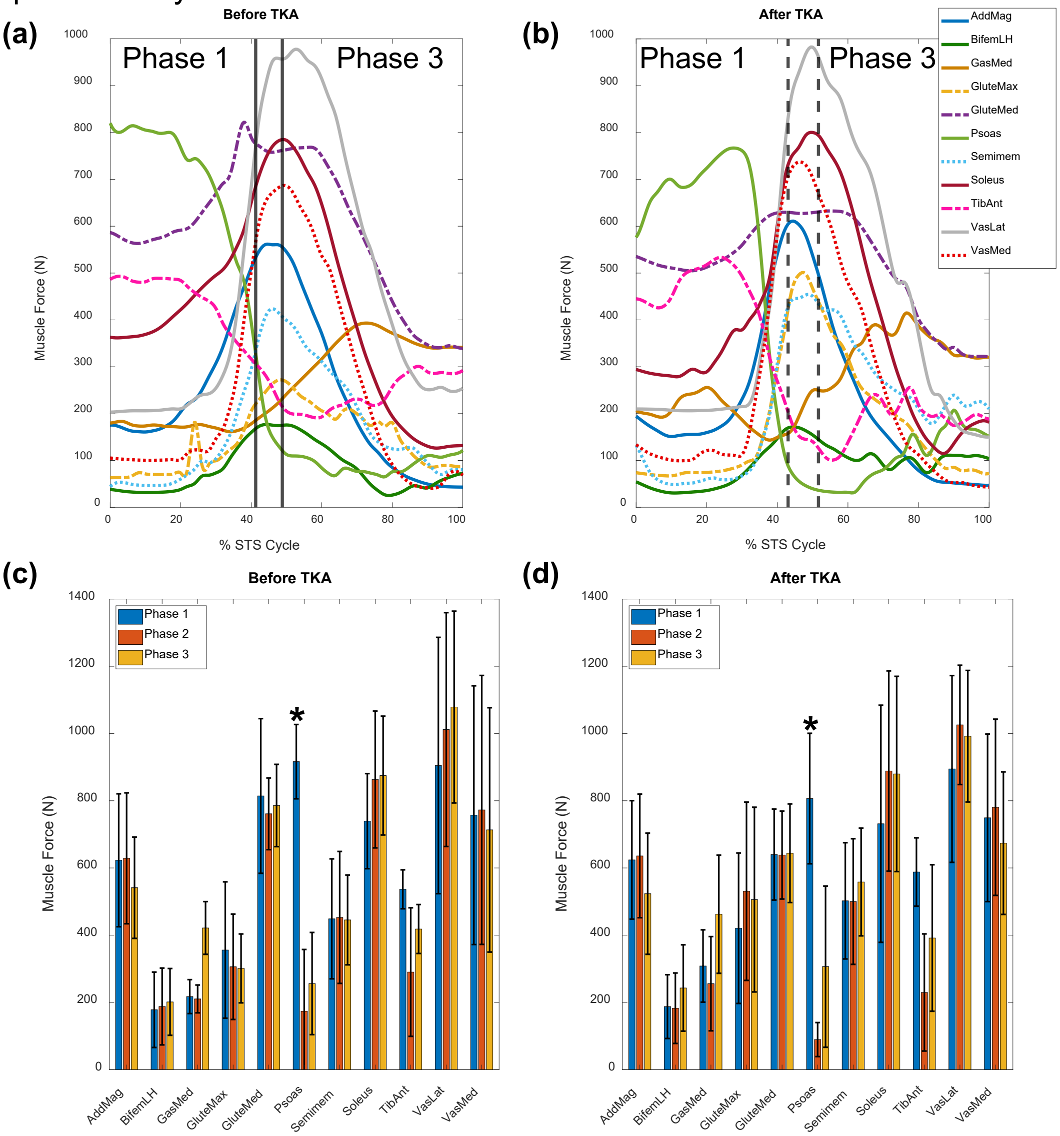

Figure S7. (a-b) Average muscle forces over the sit-to-stand (STS) cycle, for the involved limb (a) before total knee arthroplasty (TKA) and (b) after TKA. Vertical lines denote phases of the STS cycle before (solid) and after (dashed) TKA. With the exception of biceps femoris, all other muscles exhibited peak muscle forces of magnitude less than 400 N both before and after TKA. (c-d) Average peak muscle forces across all 7 participants for each phase of the STS cycle, (c) before TKA and (d) after TKA. Error bars span ± 1 standard deviation. An asterisk (*) indicates force produced for that muscle is significantly larger compared to that in other STS phases, within the same time point. Abbreviations: AddMag, adductor magnus; BifemLH, biceps femoris long head; GasMed, medial gastrocnemius; GluteMax, gluteus maximus; GluteMed, gluteus medius; Semimem, semimembranosus; TibAnt, tibialis anterior; VasLat, vastus lateralis; VasMed, vastus medialis.

Involved Limb Muscle Contributions to Center of Mass (COM) Acceleration

The involved limb's muscle contributions to COM acceleration at each time point (before and after TKA) are shown below in Figure S8 (contributions to vertical COM acceleration) and S9 (contributions to horizontal COM acceleration), with the small muscle contributions from the gluteus maximus appended to plots presented in Figures 6-7.

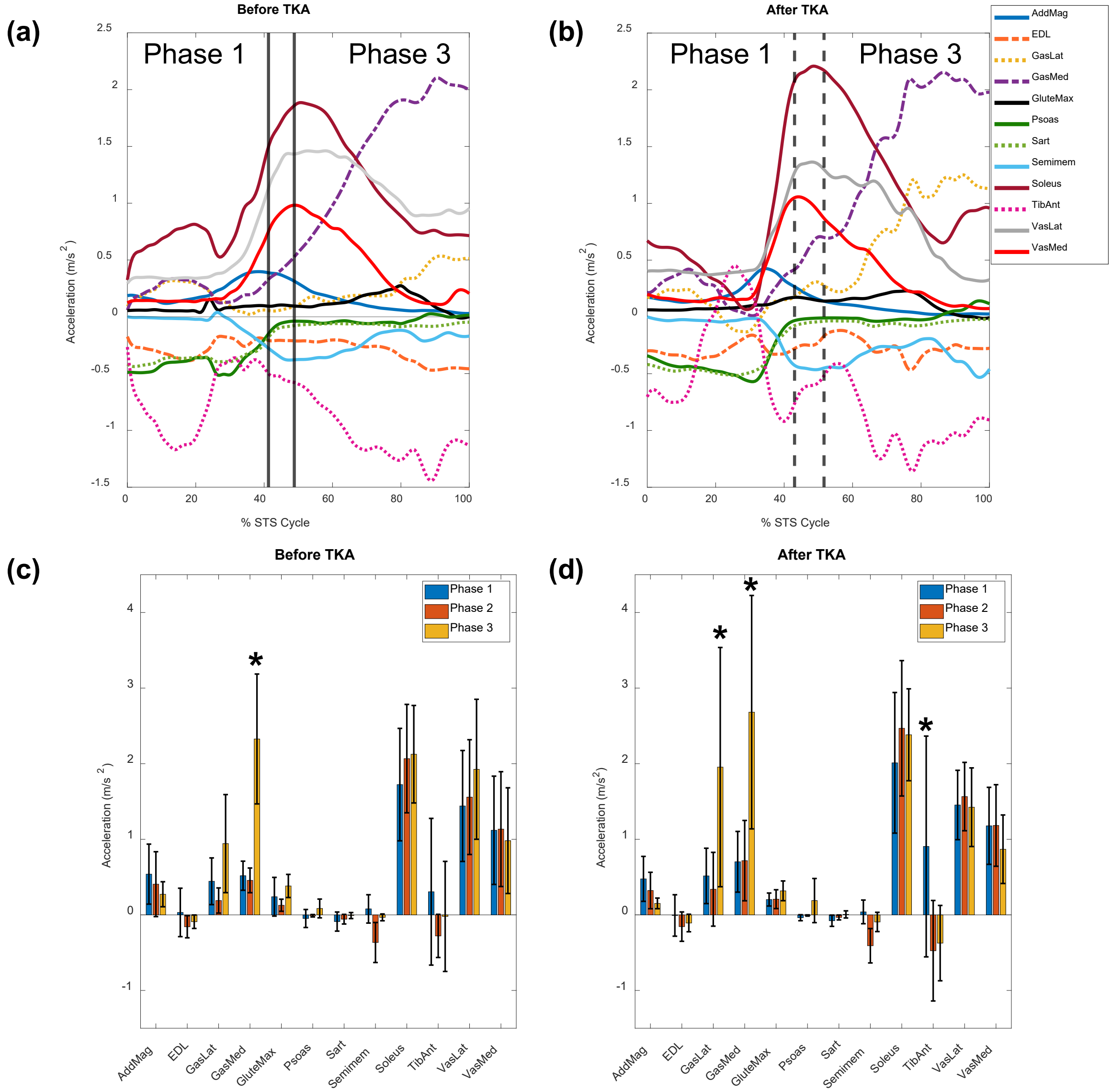

Figure S8. (a-b) Average muscle contributions to vertical center of mass (COM) acceleration for the involved limbs before (a) and after (b) total knee arthroplasty (TKA). Vertical lines denote phases of the sit-to-stand (STS) cycle. With the exception of gluteus maximus, all other muscles exhibited vertical contributions of magnitude less than 0.4 m/s^2 both before and after TKA. (c-d) Average peak muscle contributions to vertical COM acceleration across all 7 participants for each phase of the STS cycle, (c) before TKA and (d) after TKA. Error bars span ± 1 standard deviation. An asterisk (*) indicates contribution to COM acceleration for that muscle is significantly larger compared to that in other STS phases, within the same time point. Abbreviations: AddMag, adductor magnus; EDL, extensor digitorum longus; GasLat, lateral gastrocnemius; GasMed, medial gastrocnemius; GluteMax, gluteus maximus; Sart, sartorius; Semimem, semimembranosus; TibAnt, tibialis anterior; VasLat, vastus lateralis; VasMed, vastus medialis.

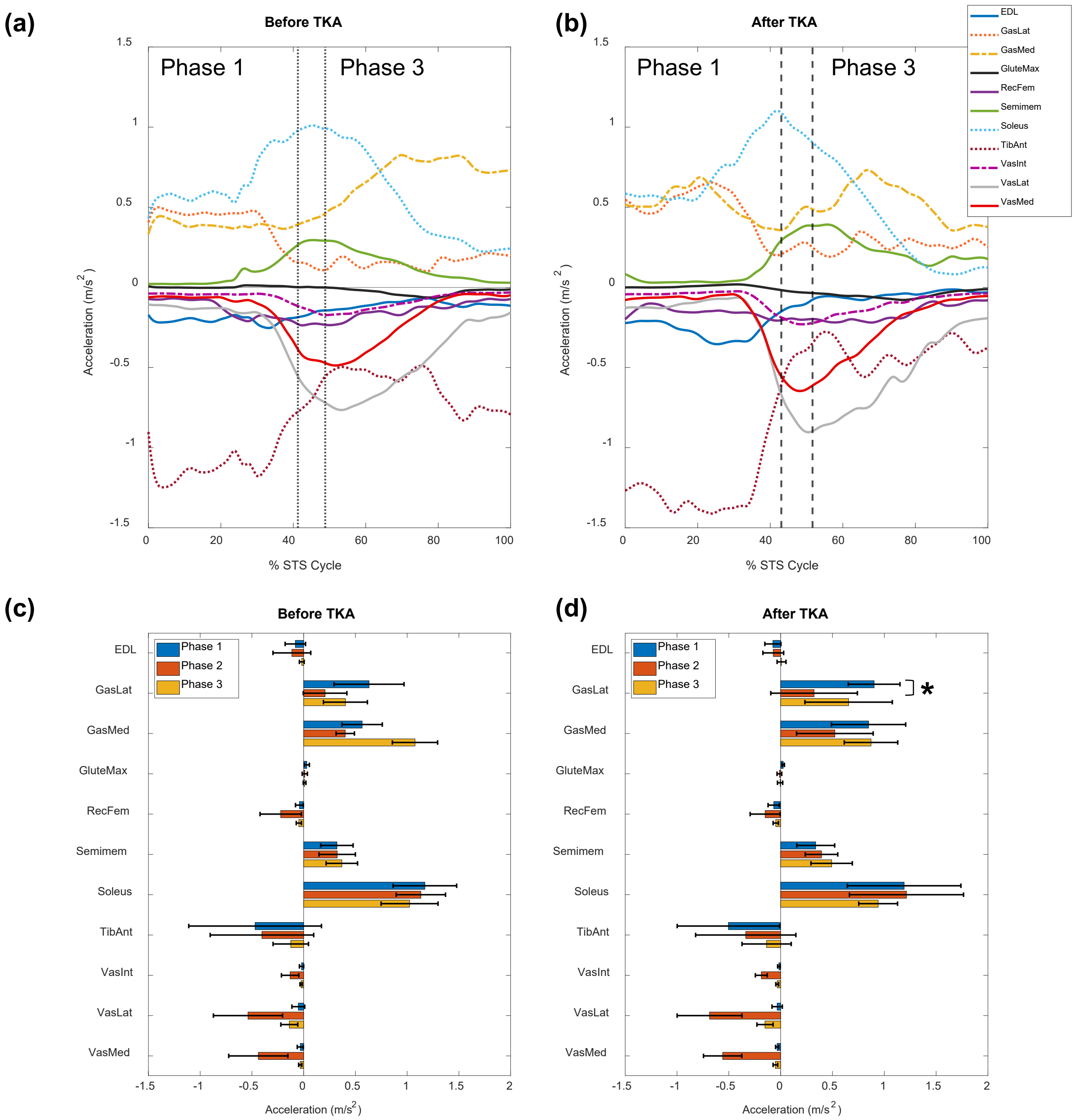

Figure S9. (a-b) Average muscle contributions to horizontal center of mass (COM) acceleration for the involved limbs before (a) and after (b) total knee arthroplasty (TKA). Vertical lines denote phases of the sit-to-stand (STS) cycle. With the exception of gluteus maximus, all other muscles exhibited horizontal contributions of magnitude less than 0.2 m/s^2 both before and after TKA. (c-d) Average peak muscle contributions to horizontal COM acceleration across all 7 participants for each phase of the STS cycle, (c) before TKA and (d) after TKA. Error bars span ± 1 standard deviation. An asterisk (*) indicates significant difference between phases for the same muscle and time point. Abbreviations: EDL, extensor digitorum longus; GasLat, lateral gastrocnemius; GasMed, medial gastrocnemius; GluteMax, gluteus maximus; RecFem, rectus femoris; Semimem, semimembranosus; TibAnt, tibialis anterior; VasInt, vastus intermedius; VasLat, vastus lateralis; VasMed, vastus medialis.

References

1. Caruthers EJ, Thompson JA, Chaudhari AMW, Schmitt LC, Best TM, Saul KR, et al. Muscle Forces and Their Contributions to Vertical and Horizontal Acceleration of the Center of Mass During Sit-to-Stand Transfer in Young, Healthy Adults. J Appl Biomech. 2016;32: 487–503. doi:10.1123/jab.2015-0291

2. Schenkman M, Berger RA, Riley PO, Mann RW, Hodge WA. Whole-Body Movements During Rising to Standing from Sitting. Phys Ther. 1990;70: 638–648. doi:10.1093/ptj/70.10.638

3. Kumar D, Manal KT, Rudolph KS. Knee Joint Loading during Gait in Healthy Controls and Individuals with Knee Osteoarthritis. Osteoarthr Cartil OARS Osteoarthr Res Soc. 2013;21: 298–305. doi:10.1016/j.joca.2012.11.008

4. Watanabe T, Koga H, Katagiri H, Otabe K, Nakagawa Y, Muneta T, et al. Coronal and sagittal laxity affects clinical outcomes in posterior-stabilized total knee arthroplasty: assessment of well-functioning knees. Knee Surg Sports Traumatol Arthrosc. 2020;28: 1400–1409. doi:10.1007/s00167-019-05500-8

5. Freisinger GM, Hutter EE, Lewis J, Granger JF, Glassman AH, Beal MD, et al. Relationships between varus–valgus laxity of the severely osteoarthritic knee and gait, instability, clinical performance, and function. J Orthop Res. 2017;35: 1644–1652. doi:10.1002/jor.23447

6. Renaud A, Fuentes A, Hagemeister N, Lavigne M, Vendittoli P-A. Clinical and Biomechanical Evaluations of Staged Bilateral Total Knee Arthroplasty Patients with Two Different Implant Designs. Open Orthop J. 2016;10: 155–165. doi:10.2174/1874325001610010155

7. Uhlrich SD, Jackson RW, Seth A, Kolesar JA, Delp SL. Muscle coordination retraining inspired by musculoskeletal simulations reduces knee contact force. Sci Rep. 2022;12: 9842. doi:10.1038/s41598-022-13386-9

8. Silder A, Whittington B, Heiderscheit B, Thelen DG. Identification of passive elastic joint moment–angle relationships in the lower extremity. J Biomech. 2007;40: 2628–2635. doi:10.1016/j.jbiomech.2006.12.017

9. Hicks JL, Uchida TK, Seth A, Rajagopal A, Delp SL. Is My Model Good Enough? Best Practices for Verification and Validation of Musculoskeletal Models and Simulations of Movement. J Biomech Eng. 2015;137. doi:10.1115/1.4029304
